# A Virtual Reality–Assisted Cognitive Behavioral Therapy for and With Inuit in Québec: Protocol for a Proof-of-Concept Randomized Controlled Trial

**DOI:** 10.2196/40236

**Published:** 2023-05-24

**Authors:** Quinta Seon, Noor Mady, Michelle Yang, Maharshee Karia, Myrna Lashley, Claudia Sescu, Maud Lalonde, Stephen Puskas, Joy Outerbridge, Echo Parent-Racine, Catherine Pagiatakis, Liliana Gomez-Cardona, Di Jiang, Stéphane Bouchard, Outi Linnaranta

**Affiliations:** 1 Department of Psychiatry McGill University Montreal, QC Canada; 2 Douglas Mental Health University Institute Montreal, QC Canada; 3 Interdisciplinary School of Health Sciences University of Ottawa Ottawa, ON Canada; 4 Ivirtivik Centre Montreal, QC Canada; 5 Makivik Corporation Montreal, QC Canada; 6 Ullivik Centre Montreal, QC Canada; 7 National Research Council Montreal, QC Canada; 8 Department of Psychoeducation and Psychology Université du Québec en Outaouais Outaouais, QC Canada; 9 Finnish Institute for Health and Welfare Helsinki Finland

**Keywords:** Inuit health, randomized controlled trial, emotion regulation, virtual reality, cognitive behavioral therapy, co-design, biofeedback, cultural adaptation, Indigenous

## Abstract

**Background:**

Emotion regulation is an ability related to psychological well-being; when dysregulated, individuals may have psychiatric symptoms and maladapted physiological responses. Virtual reality–assisted cognitive behavioral therapy (VR-CBT) is an effective psychotherapy to target and strengthen emotion regulation; however, it currently lacks cultural sensitivity and can be improved by adapting it to the cultural context of service users. During previous participatory research, we co-designed a culturally adapted cognitive behavioral therapy (CBT) manual and 2 virtual reality (VR) environments to function as a complement to therapy (VR-CBT) for Inuit who would like to access psychotherapy. Emotion regulation skill building will occur in virtual environments that have interactive components such as heart rate biofeedback.

**Objective:**

We describe a protocol for a proof-of-concept 2-arm randomized controlled trial (RCT) with Inuit (n=40) in Québec. The primary aims of this research are to investigate the feasibility, benefits, and challenges of the culturally adapted VR-CBT intervention versus an established VR self-management that is available commercially. We will also investigate self-rated mental well-being and objective psychophysiological measures. Finally, we will use proof-of-concept data to identify suitable primary outcome measures, conduct power calculations in a larger trial for efficacy, and collect information about preferences for on-site or at-home treatment.

**Methods:**

Trial participants will be randomly assigned to an active condition or active control condition in a 1:1 ratio. Inuit aged 14 to 60 years will receive a culturally adapted and therapist-guided VR-CBT with biofeedback or a VR relaxation program with nonpersonalized guided components over a 10-week period. We will collect pre- and posttreatment measures of emotion regulation and biweekly assessments over the treatment and at 3-month follow-up. The primary outcome will be measured by the Difficulties in Emotion Regulation Scale (DERS-16) and a novel psychophysiological reactivity paradigm. Secondary measures include psychological symptoms and well-being via rating scales (eg, anxiety or depressive symptoms).

**Results:**

As this is the prospective registration of an RCT protocol, we do not yet report any results from the trial. Funding was confirmed in January 2020, and recruitment is expected to start in March 2023 and is set to finish in August 2025. The expected results are to be published in spring 2026.

**Conclusions:**

The proposed study responds to the community’s desire for accessible and appropriate resources for psychological well-being, as it was developed in active collaboration with the Inuit community in Québec. We will test feasibility and acceptance by comparing a culturally adapted, on-site psychotherapy with a commercial self-management program while incorporating novel technology and measurement in the area of Indigenous health. We also aim to fulfill the needs for RCT evidence of culturally adapted psychotherapies that are lacking in Canada.

**Trial Registration:**

International Standard Randomized Controlled Trial Number (ISRCTN) 21831510; https://www.isrctn.com/ISRCTN21831510

**International Registered Report Identifier (IRRID):**

PRR1-10.2196/40236

## Introduction

### Emotion Regulation and Mental Health

Emotion regulation is a set of skills and competencies that individuals have to a variable extent: strong emotion regulation skills can facilitate mental health. When those skills are dysregulated, the difficulties related to emotion regulation are implicated in various psychological complaints and disorders [1]. Biosignals such as heart rate, skin conductance, and diastolic blood pressure response are also phenotypical in populations with difficulties in emotion regulation, indicating multiple levels of involvement in the health of individuals [2]. Some psychotherapies exert to large effect, decreasing dysregulation and increasing the ability to regulate emotions [3-6]. Improvements in emotion regulation during psychotherapy are associated with decreases in anxiety, depression, substance use, and other psychopathologies [7]. Moreover, emotion regulation is robustly and longitudinally associated with psychological resilience [8] and associated with life satisfaction, positive affect, and other well-being indicators with a small to moderate effect size [9].

### Enhancing Emotion Regulation Interventions With Virtual Reality and Biofeedback

Virtual reality (VR)–assisted mental health interventions effectively increase mental well-being, including building the ability to regulate emotions [10-12]. VR is an interactive, computer-generated, 3D world of images, videos, and sounds delivered through a VR headset system. Combined with the efficacy of cognitive behavioral therapy (CBT), v*irtual reality–assisted cognitive behavioral therapy (VR-CBT)* has several therapeutic advantages, including increased interactivity of the therapy and patient participation. Immersive visuals can create a holistic approach to therapy with mind-body exercises (eg, biofeedback integrated with VR). Therapists can present clients with enhanced skill-building scenarios and modulate elements in the environment for the personal needs of the client. For instance, the varying intensity of the exposure can be shown to the client through visual aids that reflect their physiological states [13]. *Biofeedback* is a process of rapidly demonstrating physiological metrics to an individual, sometimes with the goal of changing thoughts, behaviors, or physiology [14].

### Integrating Inuit Culture, Community Needs, and Technology

In addition to therapeutic advantages, combining VR and CBT presents a unique opportunity for the advancement of cultural sensitivity in treatment. Up to 60% of Inuit live in urban centers, and many experience adversities in health [15]. Inuit residing in urban centers reported only fair or poor access to health care [16], and in Montreal, Indigenous peoples reported trauma, anxiety, and depression to be some of their primary concerns to be addressed [17]. The increased mental health risks and lack of culturally sensitive resources for First Nations, Métis, and Inuit are part of the complex consequences of ongoing settler colonialism. The mental health impacts of colonialism can be intergenerational, with adverse impacts documented into the second and possibly third generations [18,19]. The Indigenous historical and intergenerational trauma also stresses the importance of development and research with the aim of increasing the knowledge base toward strengthening resilience of the communities and their members.

Because of its relatively low cost, flexibility, and immersion, VR has been used in Indigenous contexts, such as in art, storytelling, culture, language, and transferring cultural heritage [20-23]. There is interest in digital technology solutions for Indigenous health and mental well-being; however, most follow a format of conventional psychotherapy via telemedicine [21,24]. VR could offer opportunities for access to mental health care resources for populations affected by health inequities, delivering specialized content at a low cost and in many locations.

### Co-design and Collaboration With Inuit in Québec

#### VR-CBT

To expand access to mental well-being resources for Inuit in Québec, we will evaluate the efficacy of 2 emotion regulation–based VR interventions in a proof-of-concept randomized controlled trial (RCT). Our team developed the VR-CBT with experts in psychotherapy, VR, and Inuit health and culture, including an Inuit advisory committee. The advisory committee was composed of Inuit and non-Inuit individuals, health and social services providers, and Inuit representational organizations. The advisory committee included both women and men. We consulted Inuit representational organizations before embarking on this project with the aim of naming research priorities and building a collaboration toward those goals. We then collaborated to culturally adapt the study procedures and materials. The process of culturally adapting the treatment manual and other materials will be described further in Gomez Cardona et al, *in preparation*.

#### VR for Mental Well-Being: Self-management

Another application that is part of the study is Calm Place—a commercial VR program previously developed by Mimerse that involves guided relaxation and meditation techniques. The application has been used in clinical settings and is rooted in nature’s effects on well-being [25]. This intervention was chosen in consultation with the advisory committee based on the unacceptability of the waitlist or nonactive controls and similar therapeutic goals (eg, emotion regulation through guided activities).

### Research Aims

The primary aims of this proof-of-concept RCT are to investigate the feasibility, benefits, and challenges of a culturally adapted VR intervention versus an established VR self-management program. We will assess the feasibility and validity of psychophysiological measures as objective outcomes of the interventions. In addition, we estimate the effect size of each treatment through between-group and within-group measures of emotion regulation, mental well-being, and difficulties in mental well-being (anxiety, depression, and posttraumatic stress disorder) with a longitudinal follow-up.

We work in a novel area in the literature, providing a VR intervention for an Indigenous population with a specifically therapeutic aim [24]. In addition, we will use both objective and subjective outcome measures—a novel but robust approach [2]. This novel psychophysiological approach has been validated in healthy individuals in our laboratory (Seon et al, in preparation).

## Methods

### Design

The study design is a 2-arm proof-of-concept RCT co-designed with an Inuit advisory committee. The first treatment arm (A) is a 10-week VR-CBT. An active control treatment with minimal attention; the second treatment arm (B) will be using Calm Place as a 10-week self-management. There will be no crossover between conditions, and we will use a 1:1 ratio randomization procedure. The protocol is described in accordance with the SPIRIT (Standard Protocol Items: Recommendations for Interventional Trials) guidelines for interventional trials and is detailed in Multimedia Appendix 1. Ultimately, the goal of the study is to establish the initial feasibility of the VR-CBT and the technologies used and to identify the best measures for initial efficacy.

### Setting

This trial will take place at an outpatient psychiatric institute in Québec, Canada.

### Participants

#### Sampling

Our primary *inclusion criteria* are that the participants must (1) self-identify as Inuk, (2) live in the city where the study is taking place, (3) be aged between 14 and 60 years of age, and (4) be proficient in English or French.

With exclusion criteria, we confirm safety and best fit with the study necessitating VR and psychophysiological equipment use. We exclude those who do not identify as Inuk, youth aged <14 years, and adults aged >60 years, as well as individuals who do not tolerate VR headset and study sensors. We exclude individuals with a self-reported history of psychosis or schizophrenia, with current substance abuse as measured by screens (score >8 on the Alcohol Use Disorders Identification Test C (AUDIT-C) or >3 on the Drug Abuse Screen Test), or with any change in psychoactive medications 4 weeks before screening. In addition, we exclude individuals with physical conditions that might cause risks during VR use (ie, history of cardiac condition or epilepsy). There are no exclusions based on other concomitant care.

Meeting the diagnostic criteria for any specific disorder, verified history of trauma, or trauma symptoms was not part of our inclusion and exclusion criteria, given our focus on cultural sensitivity, our chosen skills-building approach in the therapy manual, and the shared history of trauma by community members.

#### Sample Size

There is, to our knowledge, no estimate of the effect size of emotion regulation–based VR interventions among Indigenous populations, and this proof-of-concept study will be necessary for accurate estimation of power in future trials. As described in the previous section, mental health risks among Indigenous and Inuit populations differ from those among the general population, which emphasizes the need for this proof-of-concept study specifically in this population for power calculations in future work. However, a previous VR intervention for anxiety disorders among a general population sample had an estimated effect size of g=0.78 (medium) compared with minimal-attention controls and g=0.90 (large) with no-treatment controls [26,27]. To attain statistical power >90% with an alpha of *P*<.5, the sample size of n=20 in the active treatment group and n=20 in the active control group is sufficient for generalized linear models with a similar effect size (power=0.97; *df*=38; *P*=.05).

### Procedure

#### Recruitment

The research team will recruit participants who are potentially willing to participate in either of the 2 arms of the trial. Recruitment methods will include word of mouth, flyers, and posts on social media, websites, and Montreal neighborhoods. First contacts with potential participants will involve informing them about the study and screening for initial eligibility criteria (eg, aged 14-60 years, having no acute medical or psychological risks, etc). First contacts will be done to further explain the study protocol and evaluate the eligibility criteria with potential participants. This can occur over the phone, via secure email, or in person. Our eligibility criteria include screening for suicide risk and higher scores on alcohol and drug measures. This screening will occur in person during the first visit to enroll in the study or via telephone. Once eligibility and interest in the study are established either in person or via telephone, the participants can proceed to the informed consent process with our team (in person).

#### Randomization and Masking

After receiving informed consent for participation, a research assistant will use a computerized randomization generator to randomly assign participants in a simple 1:1 ratio (VR-CBT or Calm Place). Before randomization, we will show participants the treatment rationales, explaining the 2 possible study groups. They will report their preference for either treatment in a forced-choice question, rate their preference for each treatment on a scale from 1 to 10, and provide comments on why they selected this choice. This information is collected to inform future larger trials and clinical services. The treatment cannot be supplied blinded to the content. However, as the measurements are self-reports and objective measurements, we do not use blinded outcome evaluation. Once random assignments are decided, they will be kept hidden from researchers and research assistants until the participants begin the trial.

#### Ten-week VR Interventions

##### Culturally Adapted VR-CBT as the Active Condition

The culturally adapted therapy is a manualized CBT with an emotion regulation focus. The VR-CBT therapy will take place in a clinical setting and will consist of traditional therapeutic processing complemented by therapist-guided training in a VR environment. The main therapeutic strategies are stress inoculation and guided mastery with biofeedback components. Our group developed 2 VR environments (SB UQO [Université du Québec en Outaouais] cyberpsychology laboratory)—one for safe practice and the other for challenging emotion regulation skills. The first, called Snowy Place, is a snowy tundra that mimics the arctic life well known to Inuit. In developing Snowy Place, the Inuit advisory committee believed that this would be a comforting environment. The second is a small environment with many people and no children (small stressors), where the environment and elements (eg, social and situational) demand emotion regulation skills. The participant will train learned emotion regulation skills using these environments under the guidance of a psychotherapist. The VR-CBT can have interactive components where partial avatars (eg, hands of the participant) and preprogrammed avatars (eg, static or acting people in the room) are present. Multimedia Appendix 1 provides a detailed overview of the CBT therapist manual, including a session-by-session overview.

##### Calm Place as the Active Control

During the use of the VR application, the Calm Place user may see flowing water, mountains, and tree cover; a tropical atoll with sand, palm trees, and the ocean; or an arctic scene with large pines, snow cover, and large stones. In Calm Place, each virtual environment offers applications [25,28], such as visual breathing aids, time and weather cycles, and meditation voice overs, with the guidance of the VR program. Here, the guidance on relaxation is nonpersonalized and is based on the selections of the user within the VR environment. Thus, though participants’ weekly choices regarding prevalence and duration of use are self-managed and done from their home, this intervention involves guided programming for guidance. The self-management program—Calm Place—does not have any avatar, social-interactive, or personalized components. The research team will contact these minimal-attention control participants weekly to check whether they use the application and to verify their safety.

#### Translation

A professional translator translated each of the self-reported measures that did not have the existing French versions. For comparability, given the small sample size, the surveys will be administered in either English or French for data collection, and the language controlled for in statistical analysis. However, each psychometric measure was translated to Inuktitut to complement the understanding of English and French surveys.

#### Data Collection

##### Week 0: Screening, Consent, and Randomization

**Figure 1:**
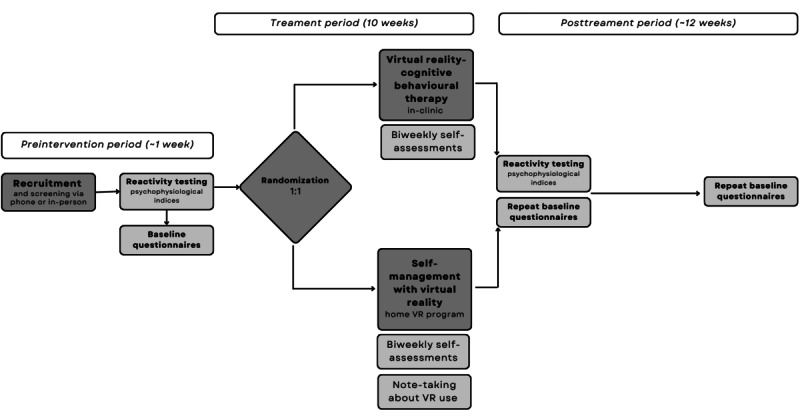
Flow of proof-of-concept study. VR: virtual reality.

Before their participation, participants will be fully informed by research assistants about their role in the study and treatment options; if willing to participate in either of the treatments, adults or guardians will sign an informed consent form (study procedures in Figure 1). The number of participants rejecting one or another treatment and thus excluded from the actual trial will be registered for evaluation of acceptance. Initial visits will involve checking eligibility and preference for treatment. We then randomly assign participants to one of the 2 VR interventions—Calm Place or VR-CBT—for a maximum of 10 weeks.

The participant study procedures from recruitment until the end of participation include a baseline measurement, a 10-week intervention period, and follow-up.

##### Week 0: Baseline Measurement of Subjective and Objective Outcomes

**Figure 2:**
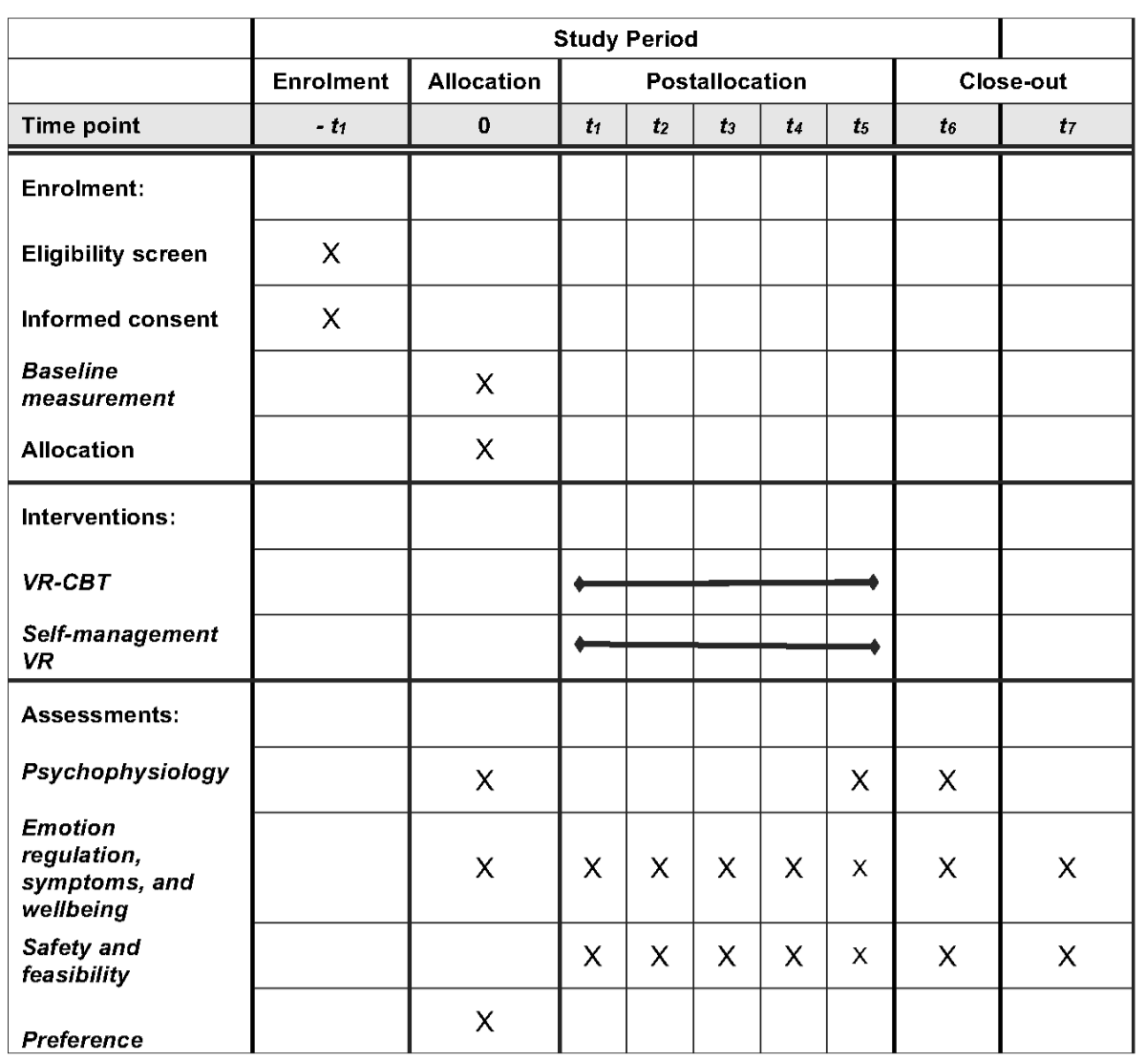
SPIRIT (Standard Protocol Items: Recommendations for Interventional Trials) flow table. VR: virtual reality, VR-CBT: virtual reality–assisted cognitive behavioral therapy.

At the first evaluation done in the days before the intervention, we evaluate the baseline subjective and objective measurements. Data will be collected and stored with REDCap (Research Electronic Data Capture) data collection software (Vanderbilt University), protecting the confidentiality of patients with research IDs. We will collect data on participants’ rest and stress psychophysiology during a 1-hour reactivity testing paradigm, where VR exposure will evoke relaxation (forest walk) or moderate stress (height exposure). Baseline measures include the Difficulties in Emotion Regulation Scale (DERS)-16 [29]; Clinical Outcome Routine Evaluation-Outcome Measure (CORE-OM) and Clinical Outcome Routine Evaluation- 10 item (CORE-10) [30,31]; Warwick-Edinburg Mental Well-being Scale (WEMWBS) [32,33]; Generalized Anxiety Disorder 7-item (GAD-7) [34]; Primary Care Screen for Posttraumatic Stress Disorder for Diagnostic and Statistical Manual 5 version (PC-PTSD-5) [35]; Patient Health Questionnaire 9-item (PHQ-9) [36]; visual analog scales for anxiety, emotional arousal, and emotional valence [37-40]; DAST-10 [41]; AUDIT-C [42]; and Severity of Dependence Scale for Cannabis (SDS-C) [43]. We will measure blood pressure using a calibrated, Health Canada–approved device. Figures 1 and 2 show the study flow diagram.

##### Weeks 1 to 10: Intervention Period and Weekly Questionnaires

During the intervention period, we will send participants biweekly questionnaires. In their final visit, participants will repeat the procedures from the first visits (visits 1 and 2). Participants will receive a small financial compensation (gift card) for each visit.

##### Week 10 to 11: Repeat Measure of Subjective and Objective Measurements

Once the intervention is complete, participants repeat the questionnaires and the reactivity testing paradigm from week zero to provide a second measurement.

##### Week 22: Postintervention Follow-up

Twelve weeks after their final visit, we will follow up with the participants on all baseline questionnaires.

### Materials

To support the delivery of the interventions and the corresponding collection of data during the trial, a sensor management system and web-based clinician portal were developed in collaboration with the National Research Council Canada (DJ and CP). The sensor management system facilitates the connection and initialization of the biofeedback sensor, eVu TPS (Thought Technology Ltd) as well as the streaming of data to the web portal. The web portal allows the management of participant sessions as well as live and postsession biofeedback data visualization. The raw biofeedback data (in CSV format) are also available through the portal to study coordinators and clinicians for postprocessing and analysis.

### Measures

#### Primary Outcome Measures

### DERS-16 Scores

The DERS-16 is a short, valid measure of emotion regulation [29,44]. Participants rate their ability to accept and be aware of their emotions, direct themselves toward their goals, control their impulses, access regulation strategies, and have emotional clarity [45]. The DERS’s psychometric properties are favorable, having moderate to very strong internal consistency (α=.69-.97) and strong retest reliability (*r*=0.88; ρI=0.85) [29, 46-49]. Correlating with anxiety, depression, stress, borderline traits, and more, the DERS-16 has good construct validity [29,47,50]. Although consistent across demographics and culturally adapted for some groups, the scale is not yet adapted for any Indigenous groups [48,51]. The full DERS is translated into French and validated [52]. Although there are no formal cutoff scores for the DERS, the English scale achieves moderate diagnostic accuracy (area under the curve [AUC]) among emotional disorders and nonsuicidal self-injury populations [53-55], and it is sensitive to changes following treatment [56-60].

### Visual Analog Scales: Anxiety, Emotional Arousal, Emotional Valence

Participants will respond to the Visual Analog Scale (VAS)-Anxiety (VAS-A), Visual Analog Scale-Emotional Arousal (VAS-EA), and Visual Analog Scale-Emotional Valence (VAS-EV) during reactivity testing. These items have favorable properties, including construct validity and sensitivity [37-40].

### Objective Measures

The participants will undergo a psychophysiological reactivity testing paradigm. This will be repeated after the end of the VR intervention for those who completed a minimum of 7 and a maximum of 10 sessions of therapy as soon as possible after the final session. We will collect and measure several metrics, including heart rate, blood volume pulse, skin conductance, blood pressure, and accelerometer data. Participants first respond to baseline subjective arousal, emotional arousal, and VAS-EV. We then begin collecting psychophysiological data with a portable finger sensor, covered with a piece of cloth for light sensitivity, by attaching it to the participant’s nondominant index finger. They fill out a survey, including several symptom scales as described during this approximately 15-minute period, and have their blood pressure taken with an ambulatory blood pressure machine. We also record a no-task seated baseline recording of sensor data for 3 minutes. Then, participants will familiarize themselves with VR in a forest walk environment with no tasks other than to walk and explore (5.5 minutes), with minimal interaction with the research team. In the next segment, participants walk a VR plank exposing them to the heights below for 5.5 minutes (instructed to walk back and forth, with no interaction). If the exercise is too challenging (eg, participant distress at a tolerable level), participants remain in the environment without walking for 2 minutes or withdraw completely (eg, intolerable distress) from the exercise. Finally, participants re-enter the same VR environment, this time with a floor rather than a plank beneath them and walk (3 minutes). The last segment is a 10-minute seated rest period. After each segment, participants report using the same visual analog scales.

#### Secondary Outcome Measures

##### PC-PTSD-5 Scores

On a scale of 5 items, the PC-PTSD-5 first verifies trauma exposure and then evaluates the presence of the 4 symptom clusters of PTSD [35]. The 5 items include symptoms such as arousal, re-experiencing, avoidance, and negative cognitions and mood. The scale has strong test-retest reliability (*r*=0.83) and is associated with important clinical variables such as suicide [35,61]. With a cutoff score of 3, the PC-PTSD-5 has strong diagnostic accuracy (AUC=0.941) [35]. The scale was translated by the Clinical Psychology and Psychotherapy Unit of the Bielefeld University into French [62].

##### WEMWBS Scores

The WEMWBS with both its 14-and short 7-item versions, measures holistic aspects of well-being. The dimensions include eudemonic aspects, hedonic aspects, psychological functioning, and subjective well-being [32,33]. Positive mental health provides an independent factor for functionality in the presence or absence of a mental illness. This scale has favorable psychometric properties. Using the WEMWBS, we can calculate mental health costs and quality-adjusted life-years [63,64]. In a study of Indigenous Australians, the Short-Form WEMWBS achieved an internal consistency of α=.69 to .88 [65]. The WEMWBS has also been evaluated in various minority and cultural groups [66].

##### GAD-7 Scores

The GAD-7 [34] is a validated self-report scale that measures anxiety presence and severity [67]. Among outpatients with anxiety and mood disorders, the GAD-7 is a reliable (*P*=.85) and sensitive (79.5%) measure with a cutoff score of 10 [68]. The GAD-7 scores have been categorized into mild, moderate, and severe anxiety, with cutoffs of 5, 10, and 15 [34,68]. Among Indigenous students in Canada, the GAD-7 achieved an internal consistency of α=.86 and correlated strongly with related constructs [69]. The scale was translated and validated in French [70].

##### PHQ-9 Scores

The PHQ-9 is a depression severity measure based on the Diagnostic and Statistical Manual Version IV [36]. In a meta-analysis of the scale’s psychometric properties, the PHQ-9 achieved optimal sensitivity and specificity with a cutoff score of 10 [71]. Among Indigenous North Americans, the scale’s psychometric properties were favorable, with an internal consistency of α=.94 [72].

##### CORE-OM, 10-Item Version

The CORE is widely used as an outcome measure in psychotherapy and has several versions, including the CORE-OM and the 10-item version CORE-10 [30,31]. The CORE-OM and CORE-10 are screening measures for psychological distress, including items for well-being, psychological symptoms, functioning, and risk. The CORE-OM has excellent internal consistency in clinical samples (α=.94) and for each of its subscales (α=.75-.94); it also has good test-retest reliability (*P*=.83) [73]. The CORE-10 with a cutoff score of 11 differentiated between clinical and nonclinical general samples with a sensitivity of .85. Its internal consistency was α=.90, and it had a reliable change index of 6 points [30]. The widely accepted French translation had validity results forthcoming from the University of Toulouse [74].

##### Feasibility: Attrition and Adherence

We will collect data about the feasibility of the treatment in the form of weekly use data (Calm Place group) and study attrition and adherence to treatment (VR-CBT group). These metrics will be assessed by the number of treatment sessions attended, completion of treatment, percentage of sessions attended, number of patients who dropped out, and when possible, reasons for dropping out, comments and notes on feasibility in diary responses, the time spent using the program, and consistency in using the program over 10 weeks. Treatment completers for VR-CBT will be defined as individuals who attend 7 out of 10 sessions, and for Calm Place, 7 reported sessions over 10 weeks. Dropouts for VR-CBT are defined as individuals missing 2 consecutive sessions of therapy without rescheduling (no-show). Noncompleters are individuals who complete <7 sessions (but attend at least 1). In the case participants are judged by the research team as *at risk* (eg, psychologically), they may also be withdrawn from the study.

### Safety: AUDIT-C, DAST-10, and SDS-C

During the treatment period, we also want to confirm that the VR-CBT is a safe pretreatment and want to observe a potential increase in the use of alcohol and drugs during the therapy. This will be done using presession evaluations with AUDIT-C, DAST, and SDS-C with a 1-week time frame. The AUDIT-C assesses alcohol consumption and drinking behaviors using 3 items [42]. The full AUDIT with 10 items has been translated and validated. Both the English and French versions of AUDIT have favorable psychometric properties, including high sensitivity and specificity (approximately 90%), and this also concerns the AUDIT-C [75-77].

The DAST is a 10-item validated self-report scale that will be used to screen for and assess drug use over the previous 12 months [41,78]. Typically, the DAST-10 achieves a sensitivity of 41% to 95% and a specificity of 68% to 99%, depending on the population and diagnostic information used [78]. No information is yet available about the validity of the French translation of the DAST.

The SDS-C[43] consists of 5 items with good internal reliability and discriminant validity, with a cutoff score of 4 [79]. No information is yet available about the validity of the French translation of the Severity of Dependence Scale [80].

### Data Analysis

#### Analyses for Rating Scales

Expecting nonnormally distributed data in a small human sample, we will conduct nonparametric tests and generate descriptive and comparative statistics to detect between-group differences for the 2 conditions, VR-CBT (A) and Active Control VR program (B), using R Statistical and Computing Software (R Core Team). We will use generalized mixed linear models to compare the 2 conditions using scores on DERS-16 across several time points (pre- and posttreatment as well as biweekly and bimonthly time points). In addition, we will describe changes in mood, anxiety, and trauma symptoms for the 2 conditions (pre- and posttreatment, as well as biweekly and bimonthly time points). The data collection for time points varies based on the administration instructions for each scale (weekly, biweekly, monthly, etc). Cohen *d* or nonparametric tests will calculate the effect size between the 2 conditions.

#### Objective Psychophysiology Data Analyses

To accurately identify peaks and not simply gradual increases in skin conductance observed over time, we will filter skin conductance data with a threshold value of 0.04 µS and average over 5 seconds [81,82]. As our study involves movement in a VR space in addition to exposure to a potential stress-inducing situation, we will filter the data with a lower limit of 40 bpm and an upper limit of 200 bpm [83]. Heart rate will also be averaged over 5 seconds. Following averaging, we will use open-source filters for preprocessing the data; then, the psychophysiological data will be processed with existing packages in MATLAB and Python. To correlate the DERS-16 and secondary outcome measures (CORE, WEMWBS, GAD-7, PHQ-9, and PC-PTSD-5) with psychophysiological data, we will employ nonparametric regression analyses in R Statistical and Computing Software. In addition, we will explore preliminary cutoff sum scores for therapeutic response by plotting receiver operating characteristic curves and conducting AUC analyses. We will also compare baseline to postintervention (second baseline) changes in psychophysiology using regression models. Cohen *d* or nonparametric tests will calculate the effect size between groups A and B.

### Ethical Approval

The protocol was approved by the Research Ethics Board of the Centre Integré de Santé et Services Sociaux de l’Ouest de l’Ile (IUSMD 21-52), and it follows the standards of the World Health Organization’s Helsinki Declaration 1975, revised in 2013. Approval for the protocol was granted by the Douglas Mental Health University Institute Ethics Review Board (IUSMD 21-52). In accordance with Tri-Council Policy Statement 2 and guidelines by the Inuit Tapiriit Kanatami and Nunavut Research Institute, the consent process involves both community and individual consent [84,85]. First, we consulted Inuit representational organizations and co-designed with community members. Before participation, we will obtain written, informed consent from all participants, their parent or guardian, or oral consent. Data will be collected and stored with REDCap data collection software, protecting the confidentiality of patients with research IDs. All publications resulting from the research will maintain confidentiality requirements by deidentifying any data. Participants will receive a small financial compensation (gift card) for each hour of in-person participation and surveys (approximately CAD $15 [US $11] per hour).

### Data Dissemination Plan

#### Respect for Inuit Ethical Standards

This research recognizes community protocols and the holistic approach to health and wellness in many Indigenous cultures. By doing that, we respect the ethics framework in Indigenous contexts, composed of principles of respect for persons and concern for Equity and Reciprocity [84]. We seek to follow and respect the principles outlined by Inuit communities in the Tri-Council Policy Statement 2 and the National Inuit Strategy on Research and the Negotiating Research Relationships with Inuit Communities guide [85,86]. These guidelines can apply to Inuit, First Nations, and Métis communities and provide structure for privacy, intellectual property, data custody, and secondary use of data. In adherence to these values, respect for people is necessary, and this is achieved through free, informed, and continued consent of the Inuit community and individual research participants [87]. We also aim toward, “research that is efficacious, impactful, and meaningful to Inuit” [85] in aligning with Inuit research priorities through consultation with representational organizations and the formation of an advisory committee. We strive to bolster social equity through the creation of materials that will belong to the Inuit community.

#### Maximized Participation of the Inuit Community

All results of this research will be validated by the community members and representational organizations and returned to them. We will communicate the final results and a report of the study to all those who participated in it and to the community organizations that would be interested. All publications resulting from the research will maintain the requirements of confidentiality and where they approve, will mention the contribution of everyone involved. The rights of coauthors of Indigenous contributors will be recognized. In any publication derived from this project, participants’ geographical localization will be mentioned as well as the Indigenous group they belong to (Inuit in Québec). This identification of participants as a group will be disclosed but not their personal information.

#### Inuit Ownership of VR Manual and Environments

The rights and permissions to use the treatment manual and VR environments belong to the Inuit community, although they are attached and available in this document. As the main intention of all products of this study is to benefit the Inuit community in Québec, the materials will not be commercialized; however, authors should be consulted before use (to be approved by the Inuit community). Any use of study materials should acknowledge the role of the advisory committee (Inuit community) in developing them and should only be used with express permission (contact SB to initiate this process).

## Results

As this is the prospective registration of an RCT protocol, we do not yet report any results from the trial. This study has been registered prospectively and is available on the web [88] (registration number ISRCTN21831510). Funding was confirmed in January 2020, recruitment is expected to start in March 2023, and is set to finish in August 2025. Expected results to be published in spring 2026.

Any major changes to the protocol will be submitted and approved by our research ethics board, documented on the trial registration platform, and justified in future publication.

## Discussion

### Summary of Objectives

This proof-of-concept study is necessary to respond to the following main concerns: (1) the limited availability of culturally acceptable versions of otherwise validated treatments [89], (2) limited number of RCTs on culturally adapted psychotherapies in Canada, and 3) the use of self-management as an active comparator. This protocol, which compares VR-CBT and Calm Place as self-management, responds to the community’s desire for culturally safe and efficient therapeutic treatments and scientific needs for further investigation into the need and efficacy of culturally adapted treatments. The Inuit community in Montreal co-designed this project with the research team based on their desire for a resilience-based mental health resource.

### Gaps in Research on Culturally Adapted Interventions

Previous evidence of increased acceptability and increased adherence of culturally adapted treatments, with a comparable efficacy to conventional psychotherapy and pharmacological interventions, is promising [90-92]. A recent systematic review of 14 studies conducted in Canada involving culturally adapted mental health treatments concluded that most interventions reduced anxiety, depression, and suicidality among First Nations, Métis, and Inuit [93]. Nevertheless, none of the 14 studies included an RCT, with most RCT evidence for culturally adapted interventions so far being conducted in the United States, low-and middle-income countries, or among refugee populations [90-93]. Furthermore, previous reports have mainly focused on clinic-based treatments or inactive controls. Although our study is randomized, we do ask about preferences between the active treatment and active control treatment and will record the number of participants excluded after accepting only one of the treatments. This will give some indication of the initial perceptions of the acceptability of the treatments.

### Implications of VR With Culturally Adapted Therapies

As technology-assisted psychotherapies advance, VR-CBT or self-management with VR may become complementary options for Indigenous health. In addition to the possibilities of remote treatment (or telemedicine), VR-CBT combines a transdiagnostic and effective treatment modality with potentially improved control and immersion into therapy [1,7]. For instance, therapists can track real-time psychophysiological responses to therapeutic challenges through biofeedback [13]. The client can participate in the therapy in any environment judged most appropriate by the therapist and client to enhance access to treatment. Lastly, Inuit in Nunavik—the northern part of Québec—often experience inconvenience and financial and emotional devastation when attempting to seek care for a health concern, as they may have to travel away from their family to the southern part of the province [94]. We explore several health technologies in the RCT, and future work includes continuing remote therapy.

### Strengths and Limitations

The process of cultural adaptation of psychotherapy was conducted to increase acceptance. The advisory committee recommended study inclusion based on sociocultural background, not trauma symptoms, to improve the cultural safety of the trial. Accordingly, inclusion is based on subjective motivation for improved mental health rather than any categorical diagnosis or subjective or objective need for treatment. In addition, the content of the psychotherapy was modified to confirm safety, according to the comments from the advisory committee. These modifications include reducing exposure and distress-producing exercises and removing the homework normally part of the therapy. A limitation is that these changes may challenge the odds of a psychotherapeutic response.

In our study protocol, we included an active control group, noting the vulnerability of the study population; this may lead to reduced between-group effects. However, only the active treatment is culturally adapted and guided by a psychotherapist, which could improve attendance and thus long-term efficacy in the active treatment group. We will observe the impact of the setting as compared with the content on acceptance while asking for the preference for therapist-guided or self-management treatment. This proof-of-concept RCT will inform us about the potential challenges or limitations of the study protocol, and we can characterize the best outcome measures and calculate power for the further efficacy trial. We consider the scientific risks acceptable, given the extreme imbalance between the need and availability for access to valid and safe treatments.

### Conclusions

The proposed study responds to the community’s desire for accessible and appropriate resources for psychological well-being, as it was developed in active collaboration with the Inuit community in Québec. We will observe preference for therapist-guided, at-clinic treatment, or at-home self-management and compare the acceptance and feasibility of a culturally adapted, VR-assisted psychotherapy with a commercial self-management program in VR. We incorporate novel technology in providing treatment and in objective outcome measurement in the area of Indigenous health. We also aim to fulfill the needs for RCTs to test the efficacy of culturally adapted psychotherapies.

## Appendix

Multimedia Appendix 1 The appendix contains the treatment manual for psychotherapists (for use contact S.B).

## Abbreviations

AUC: area under the curve
AUDIT: Alcohol Use Disorders Identification Test
CBT: cognitive behavioral therapy
CORE: clinical outcomes in routine evaluation
DAST: Drug Abuse Screening Test
DERS: Difficulties in Emotion Regulation Scale
GAD-7: Generalized Anxiety Disorder 7-item
PC-PTSD-5: Primary Care Screen for Post-Traumatic Stress Disorder Diagnostic and Statistical Manual 5 version
PHQ-9: Patient Health Questionnaire 9-item
RCT: randomized controlled trial
REDCap: Research Electronic Data Capture
SDS-C: Severity of Dependence Scale for Cannabis
SPIRIT: Standard Protocol Items: Recommendations for Interventional Trials
VR: virtual reality
VR-CBT: virtual reality–assisted cognitive behavioral therapy
WEMWBS: Warwick-Edinburg Mental Well-Being Scale.

## Data Availability

The final trial data set will be accessible to the researchers of this project. The data generated from this study are not publicly available to respect the privacy and confidentiality of participants (who are a small group that is potentially more identifiable) but are available from the corresponding author on reasonable request. The initial version of the portal (backend) that was provided to Douglas Research Institute at the beginning of the project is owned by NRC, ownership of the developments and integrations for the specific work is determined by the IP policy of Douglas Research Institute. The rights to benefit from and permissions to use the treatment manual and VR environments belong to the Inuit community, although they are attached and available in this document.
