# Supplementary material for: A Virtual Reality–Assisted Cognitive Behavioral Therapy for and With Inuit in Québec: Protocol for a Proof-of-Concept Randomized Controlled Trial
Source: JMIR Res Protoc. 2023 May 24;12:e40236. doi: 10.2196/40236 (PMC10248771; doi:10.2196/40236)
Supplement: Multimedia Appendix 1 [file resprot_v12i1e40236_app1.pdf]

Emotional Management Skills and Resilience Training with Biofeedback: Therapist Manual (version 7)

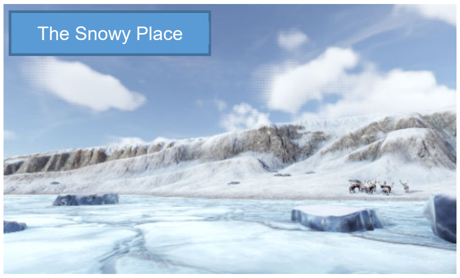

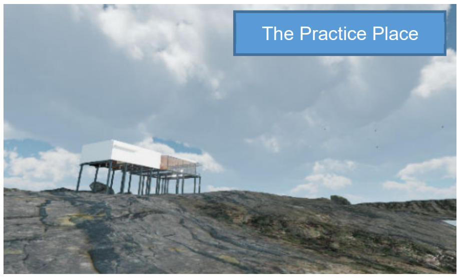

This protocol was designed to be ultimately implemented remotely in videoconference to build resilience in context with the systemic stressors afflicting the Inuit population of Nunavik. Virtual reality (VR) is used to practice and apply therapeutic strategies that can be useful to strengthen people's emotional management skills. This program is not adapted for crisis interventions. It should focus essentially on anxiety.

It is acknowledged that this manual was developed from a "Southern Canadian" perspective that adapted cognitive-behavior therapy to the Inuit culture. The current version of the treatment manual is based on a validated model of psychotherapy, but has been developed from the beginning in collaboration with our Advisory Committee of Inuit members, with input from a licensed psychotherapist living and practicing in an Inuit community, and with respect of Inuit societal values. It is important to take into consideration that Inuit are not a homogeneous group; there is significant diversity in sources of adversity (especially between generations) and in relationship with traditions. The program respects how each individual experiences its Inuit culture. The interconnectedness of mind and body, pedagogy in action, and absence of written between-session exercises are notable features of the cultural adaptations of the program.

The standardized protocol consists of 10 sessions. Each session is 60 minutes in length and scheduled weekly (or as close to weekly as possible). The majority of each session (50 minutes) is devoted to *Stress Inoculation Training* adapted to develop resilience to perceived threat emerging from adversity by using Emotional Management (EM) skills. The remaining 10 minutes of each session is spent reviewing progresses (at the beginning of the session) and planning generalization (at the end of the session). The program is built on the development of skills through collaborative work, observation and practice. The approach is closer to *Guided Mastery* principles (Bandura, 1997) rather than classical cognitive approaches. The philosophy of the program is more rooted in action done in virtual reality with the therapist to build inner-person strength than a "psychoeducational" or an "in-depth talk" therapy model. The focus is not about trauma or past distress, but on being skilled to manage current emotions.

Developed by Stéphane Bouchard, Alain Hajjar, the Cyberpsychology Lab of UQO and the research team, in collaboration with the Advisory Committee. *© 2021*

Session by Session Overview

Session	Topics / Goal
1	Establishing the ground for rapport<br>Setting goals based on a shared understanding and consensus<br>Finding personal triggers and current coping mechanisms
2-4	Relaxation to diffuse stressors: taking a step back
5	Putting thoughts and ideas in perspective – Detecting thoughts
6 - 7	Putting thoughts and ideas in perspective – Testing alternatives
8	Inner-dialogue to challenge and accept
9	Buffer session (revise or expand skills)
10	Putting it all in practice for the long term

At each session, the therapeutic skills are briefly introduced and then shown and practiced in VR. The goal of using VR is to learn by experience and practicing EM skills, so the client can progress to more challenging situations and transfer the skills to day to day situations.

Session	Sequence Within Each Session
Initial session of the program	Getting acquainted<br>Psychoeducation about how a current sense of threat can emerge from adversity and the Emotion Management (EM) model<br>Establishing intervention goals by consensus<br>Plan for the need to apply the skills in real life (between sessions)
Core of the program	Review feeling of mastery of the technique and occasions to transfer the skill since last session<br>Introduce a skill briefly<br>Practice an EM skill in a neutral context and expand on explanations<br>Practice an EM skill in a stressful context and expand on explanations<br>Consider possibilities to apply generalize skills to day to day contexts
Transition to long term gains	Review sense of mastery of the various skills<br>Explore how EM can be blended with other personal coping skills<br>Discuss emotional management over the long term and termination

Being developed for a research project, the program must be reproducible. However, it is also designed to allow therapists to slightly adapt it to the needs and pace of each individual. The therapist must manage time and efforts to ensure all content is addressed in the order planned and keep the focus on anxiety and perceived threat. The topics to work on are much focused. We recognize they are somehow narrow given the diversity of needs some people may have. However, focus is one of the key reasons why cognitive-behavior therapy (CBT) works. Yet, there is always room for fluidity in the

process. *The program is a map to journey toward a better quality of life, not a recipe to be followed blindly*.

Although Nunavik is clearly different from Nunavut, the guiding principles of the Inuit societal values and guiding principles from the Government of Nunavut (Inuit *Qaujimajatuqangit*) were considered in preparing this manual. These guiding principles are: respecting others, relationships and caring for people; fostering good spirit by being open, welcoming and inclusive; serving and providing for family or community, or both; decision making through discussion and consensus; development of skills through practice, effort and action; working together for a common cause; being innovative and resourceful; and respect and care for the land, animals and the environment.

Summary of Stress Inoculation Training (SIT) for EM

How skills will be approached in the program:

1- Introducing and explaining the skill <u>briefly</u>
2- Practicing the skill with the therapist in an easy virtual reality context
3- Practicing the skill with the therapist in a challenging virtual reality context
4- Encouraging to practice the skill in the real world between sessions

Which skill will be addressed?

1- Situational awareness – targets identification of triggers and actions
2- Relaxation training – targets current sense of threat and diffusion of crises
3- Cognitive strategies – targets threat beliefs about adversity and perceived threat
4- Inner-dialogue training – targets dealing with acute stressors
5- Closing session – targets self-appropriation, self-efficacy and long term gains

---

The Virtual Environments

The virtual environments represent two situations where participants can practice the EM skill addressed in the session. Each environment has a different purpose. In the *Snowy Place*, the client will be invited to practice EM skill and the biofeedback will illustrate visually the intensity of the stress response. The goal of the immersion in this environment is to master the skill of relaxing in a context that is meant not be stressful.

The *Practice Place* represent an imaginary place where the client can go to master the EM skills when facing more stressful content and with more complex skills. Half of the virtual space is occupied by a group of Inuit seated in circle. The other half is occupied by a stylized easel where a personalized image can be displayed. The circle must not be confused with Healing Circles. Healing Circles represent a traditional healing approach that is implemented according to several principles and procedures (Mehl-Madrona & Mainguy, 2014; Stevenson, 1999). The Talk Circle in the *Practice Place* is not used for healing per se, but to practice EM while talking in the circle. Personal images brought by the client to display on the easel are not meant for exposure to traumatic memories, but to practice EM when in the presence of a representation of a personal stressor.

Computer Interface: The *Snowy Place*

The immersion begins with a stress display of 40%. The therapist can change the sensitivity of the biofeedback as needed. A higher sensitivity is required for people who are still learning how to relax and need stronger feedback for small changes in arousal. A lower sensitivity is required for people who are getting good at relaxing and need to relax deeper to see changes in the virtual environment.

The therapist can select on the screen options to “teleport” the user to back to the start-up point, close to the caribous and to the eastern peninsula to change locations more rapidly than by walking. The Snowy Place is not intended to be stressful and no stressor can be added.

Computer Interface: The *Practice Place*

The immersion begins with a stress display of 40%. Adjust the value if needed.

The therapist can select: (a) if the man offering a drink is there or not, (b) if the client is seated in the Talk Circle of not, (c) if a personal image will be displayed or not, and (d) which of the four uploaded image will be displayed.

Menu of stressors that can be used in the *Practice Place*

Predefined	Personalized
○ A man can offer the client a drink / coffee.<br>○ It is possible to talk in the Talk Circle (about any topic, and in any language).	○ You will place on the easel a picture that represent a personal stressor for the client.<br>○ The client will be able to walk toward or away from the image on the easel, talk to the image or talk to you about it. The focus remains on EM during these actions.
You can select if the man with the drink is present or not.	You can use images brought by the client or images already on the computer. The image from the client must be in JPEG or JPG format. You can use and transfer snapshots from your cellphone if needed. Select the image **before starting** the immersion, and delete the images from the computer after the last therapy session. The name of the image must be: the **ID number of the participant_a word describing the image** (e.g., 23_dogs.JPEG).
The client can stand in the middle of the circle, or be seated as part of the circle, for the entire duration of the immersion.	The client is standing in the virtual environment and can walk in the room and on the balcony during the immersion.

About biofeedback

Biofeedback is an amazing tool to illustrate the interconnection between mind and body. It also provides explicit information on the immediate impact of relaxation exercises. As such, it is an excellent tool. However, biofeedback is double-edged sword. Using this tool can become detrimental in the treatment of anxiety disorders. When clients begin to focus more on regulating their physiological response than learning additional skills, it then becomes an avoidance strategy or a source of distraction. You will have to constantly be aware of the danger of biofeedback becoming a source of distraction. If using biofeedback focuses the client' attention away from the goal of the therapeutic exercise, then explain the situation to the client and seriously consider stop using it. Also, make sure all clients understand that biofeedback is just a tool to become more efficient in managing emotions. Its role is less important than their own skills. It is important not to give too much credit to the tool and feel empowered to manage emotions even in the absence of the tool. Therefore, it is not required to always use the biofeedback during exercises practiced in the *Practice Place*.

About cognitive restructuring and behavioral experiments

Cognitive restructuring was initially developed with a strong focus on didactic methods to identify and change dysfunctional thoughts. One important consequence of this so called "cognitive revolution" was to stir clinicians' and researchers' attention to threat appraisal, implicit information processing, and inhibitory learning principles. Since then, research has shown that, at least for anxiety disorders, cognitive restructuring using the five columns exercise is not that effective and is often difficult for clients to master. Other approaches have been successful with clients learning to ignore thoughts. And using exposure has become the key therapeutic ingredient in effective treatments of anxiety disorders. However, what remains important is the need to specifically identify what is perceived as threatening, how behavioral changes lead to cognitive changes, how to set-up exposure and behavioral experiments to create leaning opportunities that lead to long lasting emotional changes, and the relevance of cognitive-restructuring to facilitate generalization of CBT strategies to a variety of stressors and problems.

In this manual, the cognitive strategies are conceived as exercises to: (a) facilitate the identification of the sources of perceived threat (called the *Hunt for Thoughts* game), (b) putting thoughts in perspective (called the *Testing Alternatives* game), and (c) set-up personal behavioral experiments to tests beliefs and one's capacity to deal with emotions. All cognitive exercises are meant to be practiced while immersed in VR, and clients are not expected to complete the Five Columns. The clients will decide on their own how to transfer the skills to their day to day life.

Session 1 – Emotional Management and Program Goals

Introduction and review of client's goals for EM in the context of adversities (≈10 minutes)

- Introduce yourself and invite the client to introduce themselves briefly.
- Ask and address any concerns regarding the intervention. Insist on confidentiality.

Helpful tips for beginning intervention and engaging the client in the intervention

- Introduce the session by setting an informal agenda at the beginning of the session. This strategy can help provide structure to the session, which can be helpful for the client (and you): "*Today I would like to talk about …. Is this ok with you?"*
- The first session is critical for engaging the client in the intervention process and for establishing rapport (therapeutic relationship). A base rapport is an essential foundation for intervention success and establishing client's safety and trust. However, trust is gained over time, and in some contexts it may take more time to fully develop than you may be used to.
- Ensure a collaborative approach and the client's active participation in the entire intervention process (e.g., use open-ended questions to enquire about the client's current concerns; focus less on introspection and more on discussions while engaged in actions in virtual reality).
- Explain that you will the need the help of the client to help. This is a bi-directional relationship; not a situation where you have all the answers.
- Be supportive, empathetic and non-judgmental. Listen and validate the client's discomfort, concerns and experiences.
- Avoid being too didactic, technical or technique-driven. **Don't talk too much!**
- Remember to always facilitate motivation and hope.
- It may be useful to employ motivational interviewing techniques (e.g., helping the client identify the reasons for seeking intervention) with ambivalent or reluctant clients.

Psychoeducation about Emotion Regulation Model. (≈25 minutes)

- Provide a general, but brief, introduction of the intervention.
- Provide psychoeducation on the CBT model and resilience to adversity, using the model below. Remind the client this is not a therapy about being more logical (i.e., cognitive). It is about finding what is perceived as a source of threat to engage in behaviors that will contribute to resilience.

Helpful tips for providing psychoeducation

- Briefly review and normalize the client's discomforts and experiences (re-experiencing, avoidance, numbing, hyper-arousal) as well as relevant associated features (e.g., anger, guilt, depression). Focus on resilience and not on PTSD.

- Avoid *overloading* the client with too much information, technical language and jargon. Stay focused on distress, symptoms and actions
- Show the client how coping strategies are the things we have the most control over in building resilience.

Flow from Events to Emotions and Actions

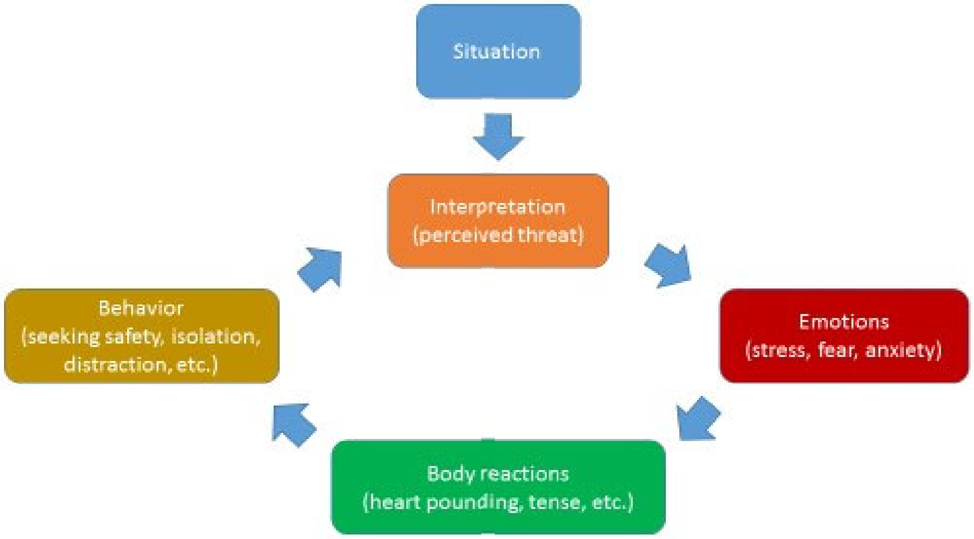

Important notes:

- Emotions can also be anger, sadness, etc. In this program, we will focus on stress/anxiety/fear.
- Behaviors to cope with situations can involve smoking, talking, drinking, becoming violent, or shutting down. We will not focus on behaviors that can cause harm to our body and mind. We will focus on behaviors that maintain fear.

Establishing Intervention Goals (≈15 minutes)

- Help client find specific (measurable) intervention goals that focus on current sense of threat (as opposed to other sources of suffering that require more focused approaches, such a hopelessness, anger, grief, school/work concerns or addiction).

**Helpful tips for setting client intervention goals**
• Ask the clients what they're looking for with this intervention.<br>• Discuss to reach a consensus on personal goals that are aligned with the goals of the program, given the research program: be empowered to manage perceived threats.<br>• Establish specific and observable goals. Ask them: "*At the end of this intervention, what would you like to be able to do <u>with your emotions</u> that would make you proud of yourself or that you can help share with other people?"*<br>• Keep the goals related to current sense of threat (i.e., not depression, etc.).

- Session 1 is the session you will talk the most. Other sessions will focus on doing things together in virtual reality. Introduce virtual reality as a tool to practice EM skills. It they are prone to motion sickness, monitor cybersickness more closely. Someone might have shown the virtual environments already, so focus on the relevance of VR more than a full demonstration.

- Show the client that the easel in the *Practice Place* is to display personalized images and it is important to start thinking about choosing them for later sessions.

Generalization and Closure of Session 1: (≈10 minutes)

- Explain the importance of practice to master skills. A therapist might use an analogy like this: "Improving your abilities in using an emotional management skill is the same as anything new, including hunting. Talking about it for hours won't change anything. Hunting at least a few times is a good start… Doing it regularly is the only way to become good at it, and maybe even teaching others how to become good too."
- For Session 1, <u>the goal is to become more aware</u> of their triggers, emotions, thoughts, and coping strategies.
- Innovation in how to practice between sessions is encouraged. It does not have to be done exactly as you would want. As long as there is generalisation to daily situations.
- Help the client prepare the generalization attempts by reviewing an example together.
- At the end of the session, assist the client to unwind before leaving the office.
- Normalize the client's reactions and remind them of potential transient side-effects of intervention (sometimes, looking at a situation can make us feel sad).
- Schedule the next session date and time.

Session 2 – EM and Relaxation Training

Review client's condition and what has been learned (≈5 to 10 minutes)

- Always follow-up on generalization attempts. Otherwise, motivation to do it will decline rapidly.
- Reinforce any effort to generalize what was learned from last week.
- Was it easy to find triggers of stress (because they already have good skills) or difficult (because they forgot, lacked the time, is wasn't clear, nothing happened) and why?
- Faced with a "Nothing happened" statement, focus on day to day stressors and the good coping strategies clients are already using. Sometimes, clients believe we have to focus on major life events but life is full of things happening every day…
- If generalization went well, take time to discuss the client's strengths and resilience and look for additional examples.
- If there was no attempt to generalize, take a few minutes to have the client examine an event that occurred between sessions and how the skill was applied.

Psychoeducation about Emotional Management (EM) (≈5 minutes)

- Ensure client understand the rationale and relevance of relaxation, then move into practice mode. In practice mode, you will always: (a) illustrate how to apply a skill, then (b) guide the client in building their confidence in using it. Reinforce small steps, encourage exploration, and be patient as perceived self-efficacy builds in.

Helpful tips for explaining the intervention rationale

- To facilitate understanding of the intervention rationale, the therapist must provide this information in the context of the client's own life experiences.
- Keep technical jargon to a minimum. Here is an example of how the therapist could describe this intervention: "I would like to show you a series of skills which people really find effective to manage emotions and become even more resilient. After I briefly show you a skill, we will practice it together in virtual reality. We will then find situations for you to practice it in the real world as your personal exercise for the week. Although we will focus on current sources of threat, these skills can also be used to help you manage other emotions like sadness, anger, disgust, shame, etc."
- The most important thing is to ensure that the client leaves the session with an experiential understanding of how to use relaxation.
- Sometimes it can be helpful to introduce the rationale of EM by asking the client, "How did you manage emotions observed last week exercises? "You can then use their examples to introduce EM.

Discussion about the impact of psychotherapy

- As part of the informed consent process, it is important to discuss the impact of psychotherapy. A therapist should mention: "Although there is no guarantee, we hope that many people will benefit from the program we are testing now with virtual reality. At the same time, it can be a challenging intervention and some people experience a temporary increase of anxiety and other symptoms, particularly when talking about your emotions. If at any time you have any questions or concerns about the intervention, please let me know. Do you have any questions about that now? Are you ready to get started?"
- Always provide an opportunity to let the client ask questions or discuss concerns about the intervention throughout the program.

Introduction to Relaxation Training (≈10 minutes: 5 to talk + 5 <u>to do it yourself</u>)

<u>Information for therapists:</u> *Jacobson's Relaxation Technique* is a type of strategy that focuses on tightening and relaxing specific muscle groups in sequence. It is also known as *Progressive Relaxation Therapy.* By concentrating on specific areas by tensing and then relaxing them, you can become more aware of your body and physical sensations.

The Tension Phase is used only to become better at noticing tension and the contrasts with relaxation. Once relaxation is becoming mastered, the *Tension Phase* will be abandoned to use only relaxation. *The Relaxation Phase* will be paired with the reduction of muscle tension occurring when we exhale. This release in tension and relaxation can then be applied in contexts that are progressively more stressful. Post relaxation, new actions can then be appraised with less emotions.

- Relaxation Training: *Jacobson's Relaxation Technique* (simplified).
- Introduce it as a tool to diffuse intense stress or situations. Approach this technique not ONLY as way to reduce current physiological stress levels, but also as an OPPORTUNITY that lead to replacing unwanted/destructive behaviors (*i.e. this will help you prevent other unwanted behaviours such as safety seeking behaviours, violence, destructive behaviours, etc.*).

- Present the two virtual environments on the computer monitor, and the biofeedback sensors. The *Snowy Place* is the first environment you will use to practice the skills together in a context that is easy.

How to do relaxation:

1- At home: sit down comfortably with your hands on your sides or on your lap.
2- Pick two to four *Tense Areas* in your body. Identify them explicitly.
3- Take in 3 big breaths from your belly (abdomen).
4- Pick a first *Tense Area* in your body and follow these steps:
    a. Bring your attention to the muscles in that area.
    b. **Tighten** your muscles gently, but don't strain.
    c. **Notice the tension** for a few moments (3-5 seconds)
    d. **Release the tension** and notice the relaxation appearing.
    e. **Repeat** and become more and more aware of the difference between the muscles when they're tensed and when they're relaxed.
    f. **As you exhale**, let even more tension go away every time.
5- Move on to another tense area and repeat.

Helpful tips for introducing the Jacobson's Relaxation Technique in 5 min.

- Typically, the *Jacobson's Relaxation Technique* is done as a whole-body technique and progressively from head to toes. This makes it a long process (20-25 minutes). Our program focuses on more localized technique and combines the effect of exhaling and releasing tension. **To begin**, ask the client to identify where they usually feel stress in their body and what muscles feel tense at the moment. Then pick 2-4 tense areas to apply relaxation locally.
- This technique should be viewed purely as a way **to reduce physical tensions** in the body and not as a focused meditation technique to *clear the mind.* That being said, you can mention to the clients that it is normal (and expected) they will have intrusive thoughts while doing this relaxation technique. Their task at that moment is to bring their attention back to the tensions in their body.
- Clients may feel restless or frustrated when first practicing relaxation. If this is the case, keep the exercise short and reinforce their efforts.
- As the skill gets mastered and applied in day to day setting, only letting go or pairing exhaling and relaxing will become sufficient.

Illustrating Relaxation While the Therapist is in VR With the Biofeedback

- As you are showing the technique to the client by applying yourself during 5 minutes, use the VR environment and the biofeedback to illustrate how your body react to the technique while you are in the *Snowy Place*.
- Point out how the blizzard increases as you are getting tense and disappear as you are getting relaxed.
- When relaxed, you can hear the snow under your boots. As stress increases, the snow falls much more rapidly, the wind picks-up in intensity, and it becomes harder to see your surroundings. The weather changes very rapidly, just as fast as your tension is changing.

Practicing Relaxation Training in an Easy VR Context (≈25 to 30 minutes)

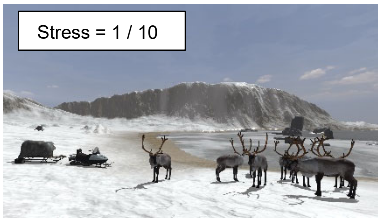

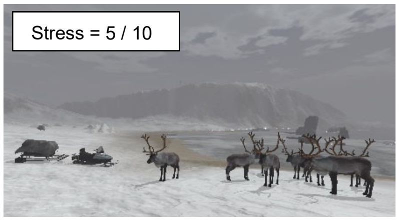

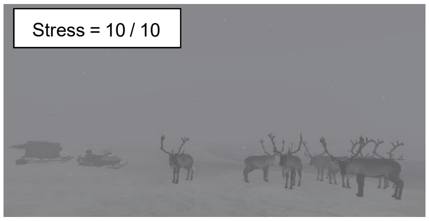

Guide the client in relaxing and reducing the intensity of the blizzard in the *Snowy Place*.

The client can explore and walk anywhere in the VR environment.

<u>**Computer Interface**</u>:

The immersion begins with a stress display using the full sensitivity scale. You can adjust the sensitivity by reducing / expanding with the mouse the minimum and maximum values.

<table>
<tr><td>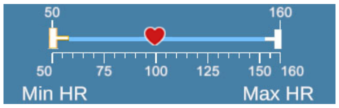
</td><td colspan="2"><- <u>Less sensitive</u> to variations in hear rate, because HR has more room to change from Min (0%) to Max (100%).</td></tr>
<tr><td>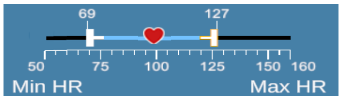
</td><td colspan="2"><- <u>More sensitive</u> to variations in heart rate, because small changes in HR represent larger change from Min (0%) to Max (100%).</td></tr>
<tr><td>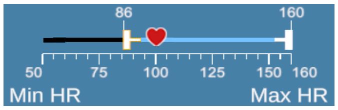
</td><td>You can also make it more sensitive by moving only the Min or Max values</td><td>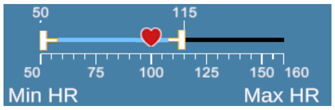
</td></tr>
</table>

The therapist can use the icons to change locations more rapidly than by walking.

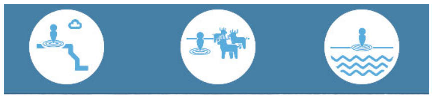

Generalization and Closure of Session 2: (≈10 minutes)

- Explain the importance of practice to master skills.
- Always make sure the client understands the point of generalizing between sessions the skills that were developed today.
- For Session 2, <u>the goal is to relax when already slightly calm</u> (e.g., when walking, taking a break or out smoking).
- Innovation in how to practice between sessions is encouraged. It does not have to be done exactly as you would want. As long as there is generalisation to daily situations.
- Help the client prepare the generalization attempts by reviewing an example together.
- <u>Explore with the client which images of personal stressors they can bring (between 1 and 4) and how it will be done (USB stick, email, physical objects, etc.).</u>
- At the end of the session, assist the client to unwind before leaving the office.
- Schedule the next session date and time.

Sessions 3 and 4 – EM and Relaxation Training in a More Challenging Context

Review client's condition and what has been learned (≈10 minutes)

- Always follow-up on generalization attempts. Otherwise, motivation to do it will decline rapidly.
- Reinforce any effort to generalize what was learned from last week.
- Was it easy to relax (because they already have good skills) or difficult (because they forgot, lacked the time, is wasn't clear, nothing happened) and why?
- Faced with a "Nothing happened" statement, focus on day to day stressors and the good coping strategies clients are already using. Sometimes, clients believe we have to focus on major life events but life is full of things happening every day…
- If generalization went well, take time to discuss the client's strengths and resilience and look for additional examples.
- If there was no attempt to generalize, take a few minutes to have the client examine an event that occurred between sessions and how the skill was relevant or applied.
- Get the personal images from the client, and save on the computer them according to the convention (Research ID number _ a word describing the image). If they did not bring images, select images from our list of generic images.

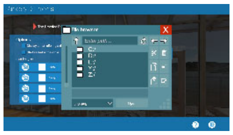

Practicing Relaxation Training in an Easy VR Context (≈5 minutes)

- We will do it for 5 minutes in the *Snowy Place*. "Last week, we practiced with relaxation in a relatively easy environment. This week, we will practice in a more challenging context. " It is ok if the client requires more time in the *Snowy Place*.

- Then we will do it for 35 minutes in the other VR environment.

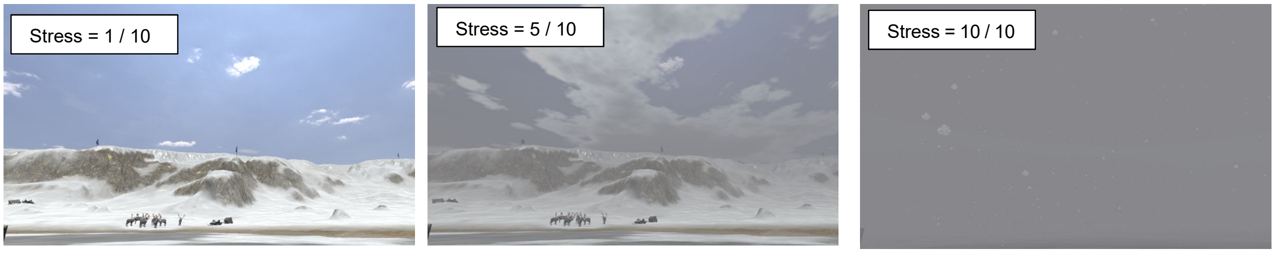

Guide the client in relaxing and reducing the intensity of the blizzard in the *Snowy Place*.

Practicing Relaxation Training in a More Challenging VR Context (≈35 minutes)

- Introduce the *Practice Place* virtual environment to the client **as a place that does not exist,** but will be used over the program to practice EM in more challenging contexts. This is the area where we will play "EM games" every week.

- First, use the environment without adding any stressor. Guide and support the client in relaxing (reducing the intensity of the blizzard and boosting the Qulliq lamp) in such a strange place. Encourage them to walk around while staying relax.
- Help the client notice how the blizzard is increasing in intensity outside AND the size and intensity of the Qulliq lamp that is visible in his right field of view. The blizzard provides the same kind of biofeedback as in the *Snowy Place*. The Qulliq lamp is used to display the biofeedback inside the room as well.
- When the client has become familiar to the *Practice Place* and is ready to add stressors, let the client immerse in VR and discuss which stressor would be acceptable to add in the *Practice Place* so they can practice relaxation. Base your selection of stressors on what you have learned from the client so far as well as the menu of available stressors in the program.

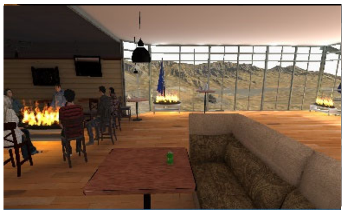
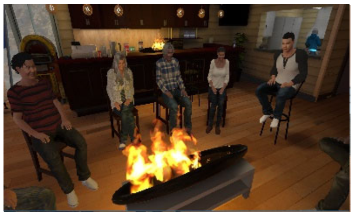
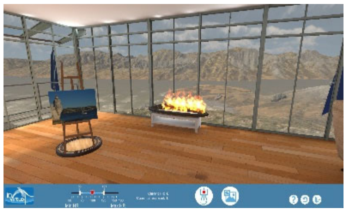

<u>Computer Interface</u>:

The immersion begins with a stress display using the full sensitivity scale. Adjust the sensitivity, if needed, by reducing or expanding the minimum and maximum values.

The therapist can select: (a) if the man offering a drink is there or not, (b) if the client is seated in the Talk Circle of not, (c) if a personal image will be displayed or not, and (d) which of the four uploaded images will be displayed.

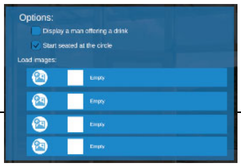

- **Menu of stressors that can be used in the *Practice Place***

<u>Predefined</u>

- A man can offer the client a drink / coffee.
- It is possible to talk in the Talk Circle (about any topic, and in any language). Highlight the goal of managing emotions and stress. This is <u>not</u> a Healing Circle. If clients want to use is as a Healing Circle, respect their decision, and agree if you can intervene to guide them in EM while they are talking. Focusing on EM should be the common goal.

<u>Personalized</u>

- You will place on the easel a picture that represent a personal stressor for the client.
- The client will be able to walk toward or away from the image on the easel, talk to the image or talk to you about it. The focus remains on EM during these actions.

- The first time the client is in the *Practice Place*, discuss which image would be appropriate and select it.

- On other occasions, use personal images the client brought with them.

- If needed, you can use ***<u>generic</u>*** images already available on the computer.

- Guide and support the client in relaxing (reducing the intensity of the blizzard and Qulliq lamp) despite the stressors. Reinforce every success.

Generalization and Closure of Sessions 3 and 4: (≈10 minutes)

- Explain the importance of practice to master skills. Always make sure the client understands the point of the take-home exercise.
- For Sessions 3 and 4, <u>the goal is to relax in more difficult situations</u>.
- Innovation in how to practice between sessions is encouraged. It does not have to be done exactly as you would want. As long as there is generalisation to daily situations.
- Help the client prepare the exercise by reviewing an example together.
- At the end of the session, assist the client to unwind before leaving the office.
- **<u>Plan with the client which pictures to bring next week</u>** (between 1 and 4) and in which format (JPEG, an object you will need to take a picture of).
- Remember that for Session 4 the images need to be more stressful and in later sessions the stressors will be used to work on thoughts. Make sure you have images that will be relevant for the work to be done.
- Schedule the next session date and time.

The biofeedback is expressed by the warmth / intensity of the Qulliq lamp and the intensity of blizzard visible by the windows, as illustrated below.

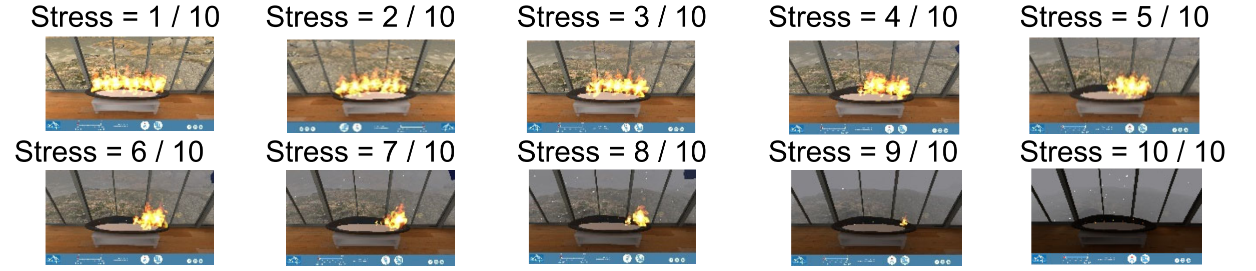

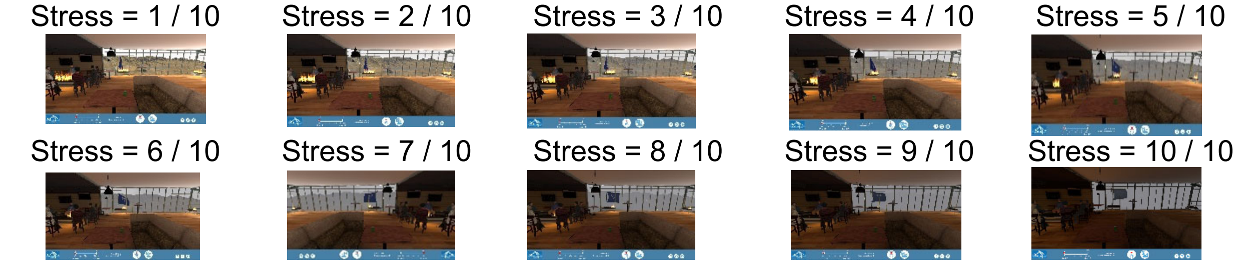

You can modify the option of the easel to stop always facing the user: 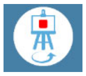

To reactivate this option, click on the icon with the green button: 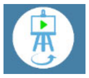

Session 5 – Putting Thoughts and Ideas in Perspective (Phase 1)

Detecting Thoughts

Review Client's Condition and what has been learned (≈10 minutes)

- Always follow-up on generalization attempts.
- Reinforce any effort to generalize what was learned from last week.
- Was it easy to relax (because they already have good skills) or difficult (because they forgot, lacked the time, is wasn't clear, nothing happened) and why?
- Faced with a "Nothing happened" statement, focus on day to day stressors and the good coping strategies clients are already using. Sometimes, clients believe we have to focus on major life events but life is full of things happening every day…
- If generalization went well, take time to discuss the client's strengths and resilience and look for additional examples.
- If there was no attempt to generalize, take a few minutes to have the client examine an event that occurred between sessions and how the skill was relevant or applied.
- Is the client able to relax directly from the *Relaxing Phase*, and while facing actual stressors?
- Depending on the level of mastery of the skill, recommend to continue practicing until confidence is strong or applying it whenever necessary from now on.

Introduction to Cognitive Strategies (Phase 1) (≈10 minutes: 6 to talk, 4 to do it yourself by describing a personally stressful situation of your past)

- Thoughts are NOT reality, they are the first step in a sequence.
- The point it NOT that clients are not thinking correctly. The point is that thoughts can be helping or not (leading to dysfunction)
- Let's begin by identifying thoughts and then we will challenge them.
- CULTURAL SAFETY: ***Cognitive restructuring is always non-judgmental***

Cheat sheet for detecting thoughts when facing current threats in VR

- What are you I thinking right now?
- What is the worst that can happen?
- Why is the current situation worse than other situations?
- What would make the current situation worse, or better?
- What does the current situation mean to you?
- Do you have a troubling image in your mind (instead of a thought)?

Practicing Cognitive Strategies (Phase 1) in an Easy VR context (≈10 minutes).

- Use the *Snowy Place* with the biofeedback (unless it is attracting the client’s attention too much) and the sheet *The Hunt for Thoughts*.
- Start by looking at the environment and try exploring current emotions and thoughts. When the client is able to identify any emotions or thoughts, begin exploring positive emotions and thoughts experienced right now. If it is difficult to name thoughts, use the cheat sheet above. If negative emotions and thoughts are experienced, this is OK. Use examples of how you would feel in this VR environment if necessary. As the exercise progresses, look for thoughts that lead to emotions (core thoughts) as opposed to comments about the virtual environment (e.g., “It doesn’t look like a place I have been before”.) or general ideas (automatic thoughts, e.g., “It is going to be a warm day”.). '

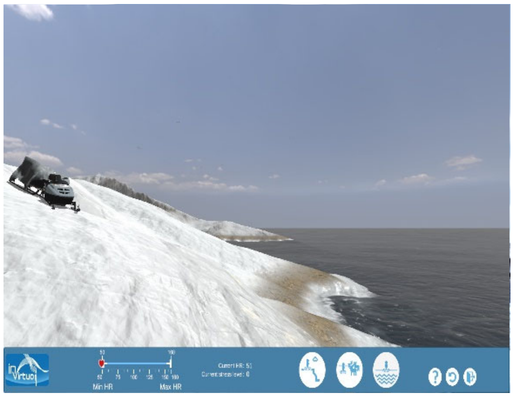

- Complete *The Hunt for Thought* sheet for the client while they are immersed in VR and discuss it during or after the immersion.
- Some people prefer to list Thoughts before Emotions in The Hunt for Thoughts (because it is thoughts that actually leads to emotions), and other prefer listing Emotions first (because they experience emotions first). This is a matter of client’s preference.
- Be mindful that client may have experience significant adversity, often systemic and generational, and often from people saying they want to help. Do not question these issues. Leave space to express anger and grief, and then focus on here and now situations that are amenable to cognitive restructuring.

Practicing Cognitive Strategies (Phase 1) in a More Challenging VR context (≈20 minutes)

- Use the *Practice Place* and *The Hunt for Thoughts*.
- Before starting the software, ask the client to identify which stressor will be used (i.e., the man, the Talk Circle, which personal image).
- Help the client identify what thoughts are hidden behind the negative emotions.
- Highlight that there may be more than one thought. The thoughts are not erroneous (i.e., they may be true), they are just not helping and lead to less resilience (i.e., dysfunctional).
- Do not judge the believability of the thoughts, unless: (a) clients are ready to move to Phase 2, or (b) thoughts are too distressing.

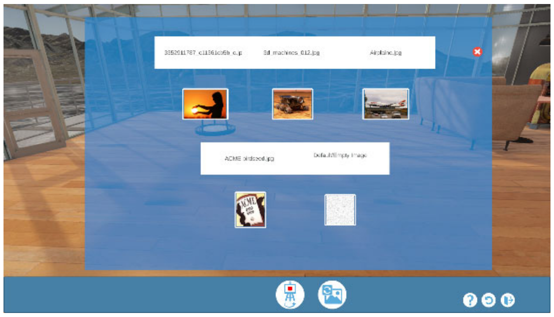

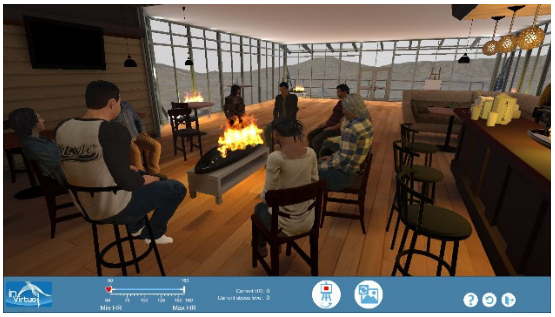

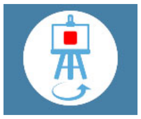

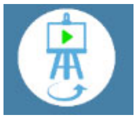

<u>Computer Interface</u>:

The immersion begins with a stress display using the full sensitivity scale. Adjust the sensitivity, if needed, by reducing or expanding the minimum and maximum values.

The therapist can select: (a) if the man offering a drink is there or not, (b) if the client is seated in the Talk Circle of not, (c) if a personal image will be displayed or not, and (d) which of the four uploaded images will be displayed.

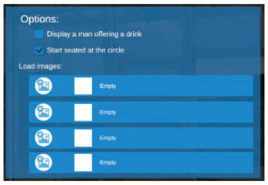

O Menu of stressors that can be used in the *Practice Place*

<u>Predefined</u>

- O A man can offer the client a drink / coffee.
- O It is possible to talk in the Talk Circle (about any topic, and in any language). Highlight the goal of managing emotions and stress. This is <u>not</u> a Healing Circle. If clients want to use is as a Healing Circle, respect their decision, and agree if you can intervene to guide them in EM while they are talking. Focusing on EM should be the common goal.

<u>Personalized</u>

O You will place on the easel a picture that represent a personal stressor for the client.

O The client will be able to walk toward or away from the image on the easel, talk to the image or talk to you about it. The focus remains on EM during these actions.

- Use personal images the client brought with them.

- If needed, you can use ***<u>generic</u>*** images already available on the computer.

Generalization and Closure of Session 5 (≈10 minutes)

- O Show how you used The *Hunt for Thoughts* while the client was in VR and invite the client to play the game *The Hunt for Thoughts* to strengthen generalization. <u>Do not make it a formal homework</u>. It is a fun game to try!
- O For Session 5, <u>the goal is to be able to find some thoughts associated with threat</u>.

Healing my Emotions Game 1: The Hunt for Thoughts (in VR)

Situation	Emotions	Thoughts

Session 6 - Putting Thoughts and Ideas in Perspective (Phase 2)

Testing Alternatives

Review client's condition and what has been learned (≈10 minutes)

- Follow-up on generalization attempts. Reinforce all efforts.
- If there was no attempt to generalize, take a few minutes to have the client examine an event that occurred between sessions and how the skill was relevant or applied.
- Follow-up on <u>relaxation</u>: is the skill mastered and applied whenever relevant?
  - ☐ If this is too difficult to be mastered by now, consider putting it aside. Some people just don't particularly like relaxation or can't relax very easily. This is not a failure, but an indication that it is time to try something else.
  - ☐ If the client is building a sense of mastery, highlight the importance to continue using it as they become better and better.
- Follow-up on <u>cognitive restructuring</u>: was it easy to play *The Hunt for thoughts* (because they already have good skills) or difficult (because they forgot, lacked the time, is wasn't clear, nothing happened) and why? Do not pay too much attention to the labeling of emotions. Focus on situations that occurred during the week in which underlying thoughts were difficult to find. Make the exercise a game of finding the cues that lead to unwanted emotions. During *Phase 1*, it is too early to challenge thoughts. For some people finding dysfunctional thoughts takes a lot of time. If the client is able to move to *Phase 2* earlier, then do not hesitate to do so. Remember: testing includes behavioral experiments, so it is an experiential and behavioral experience.
- Faced with a "Nothing happened" statement, focus on day to day stressors and the good coping strategies clients are already using. Sometimes, clients believe we have to focus on major life events but life is full of things happening every day…
- <u>Depending on the level of mastery</u> of the skill:
  - Recommend continue practicing *Phase 1* until confidence is strong, including during the session today. This means you will get behind in the program by one session, and it is fine. Remember that you have one buffer session (#9).
  - Move to *Phase 2* and add restructuring dysfunctional thoughts (Testing alternatives) today.

Introduction to Cognitive Strategies (Phase 2) (≈10 minutes: 7 to talk, 3 to do it yourself by describing a personally stressful situation of <u>your</u> past)

- Introduce the notion of putting thoughts in perspective. <u>This helps to clarify that their thoughts are not erroneous or that the client is not thinking incorrectly.</u> This examination is necessary to take a step back and analyze thoughts from a different perspective. In this phase, we put thought to the test.
- The cheat sheet list below suggests questions that people find useful to examine if thoughts pass the test of realism.

Cheat sheet for testing alternative thoughts

- Re-assess the consequences:
  - So what if it happens?
  - How will the situation end, realistically?
  - Are there other possible consequences?
  - What are the alternative explanations?
- Re-assess the likelihood:
  - What proofs do you have, pros <u>and</u> cons?
  - Is this a reliable source of information?
  - Are you using my emotions to guide your assessment?
  - What is the most likely conclusion to this situation?

- The strategy will be fully practiced in VR.
- Keep your personal example simple.
- Focus on perceived threat as much as possible.
- Help clients find the questions that work well for them.
- <u>Be mindful that clients may have experience significant adversity, often systemic and generational</u>, and often from people saying they want to help. Do not question these issues. Leave space to express anger and grief, and then focus on here and now situations that are amenable to cognitive restructuring.

Practicing Cognitive Strategies (Phase 2) in an Easy VR context (≈5 minutes)

- Use the *Snowy Place* (with biofeedback), and the *Testing Alternatives* sheet.
- If the immersion induces negative emotions, begin applying the strategy of testing alternative thoughts. If the immersion in VR induces only positive emotions, then highlight that peace and resilience is possible and that not all thoughts are dysfunctional. Also note that walking on the land represent a safe place for that client. Then transition to the other VR environment.
- Notice that *Thoughts* are now listed in the *Testing Alternatives* game (see previous week for client's preference on where to list thoughts and emotions).
- In the *Testing Alternatives* game, the percentages are mentioned only for the therapist. The clients are not expected to provide percentages. The percentage rating for the dysfunctional thoughts is before the test is applied and the second ratings of emotion's intensity pertain to after the test has been applied. This means that if the intensity of the emotion does not decrease, it is either because you are not targeting relevant thoughts, or that confidence is not high enough in the alternative thoughts (more on confidence below).
- Remember that there may be more than one dysfunctional thought and that confidence in alternative thoughts <u>will grow with time and collecting proofs</u> <u>between sessions</u>. **The focus is not on intellectual work, but on pragmatic testing.**
- Testing alternative thoughts is not the equivalent of positive thinking. Positive thinking does not help facing perceived threat. Realistic thinking does…
- In cognitive restructuring, we are not testing a person's initial thoughts as much as we are testing for alternative and more functional thoughts. This slight difference puts the focus on a quest for alternative and realistic views and strays away from judgement of thinking patterns from the client. It is also focused on action!

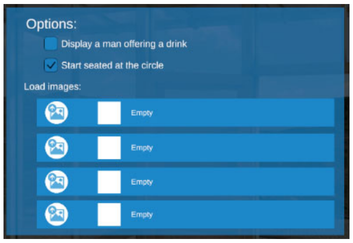

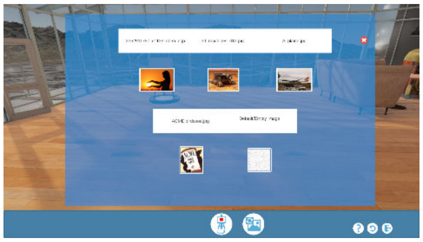
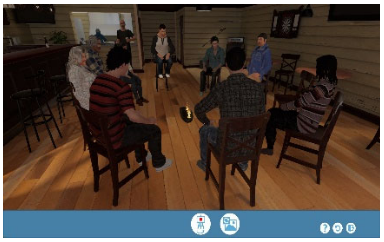

Practicing Cognitive Strategies (Phase 2) in a Challenging VR context (≈25 minutes)

- Use the *Practice Place* (with biofeedback), and the *Testing Alternatives* sheet.
- <u>Before</u> starting the software, ask the client to identify from the menu which stressor to add to the virtual environment. Agree on a progressive sequence that will maximize emotional safety and the development of resilience skills.
- Guide clients in finding alternative thoughts on their own and check if these alternative views decrease the negative emotions. Be pragmatic and objective; it is not rationalization but actual testing based on facts. Setting behavioral experiments may be needed between sessions. Complete the *Testing Alternatives* sheet is if it is game. Writing is typical of "Southern Canadians", but do not expect clients to do the same. Unless they request copies to play the game on their own.
- Complete the *Testing Alternatives* sheet for the client while they are immersed in VR and discuss it during or after the immersion.
- Adjust the number of stressors and accompany the client in their quest for testing alternative thoughts. The idea is to look for alternative thoughts and discuss how they can realistically explain the situation.
- There is no rush to proceed rapidly in the VR environment. Let the client explore the environment, process the situation, come with alternative views and assess their plausibility. Guide and coach them in the process by pointing to ideas and reinforcing progress.
- Do not aim for a profound introspection and shared emotional experiences. Aim for a collaborative process where people learn by experience and reinforced successes.

Generalization and Closure of Session 6 (≈10 minutes)

- Show how you used the *Testing Alternatives* game while the client was in VR and invite the client to play the game to strengthen generalization. <u>Do not make it a formal homework</u>. It is a fun game to try!
- For Session 6, <u>the goal is to be able to reframe (restructure) some thoughts.</u> Ideally, thoughts associated with perceived threat. The more the client can test and become able to put things in perspective, the better.

Healing My Emotions Game 2: Testing Alternatives (in VR)

Situation	Thoughts (%)	Emotions (%)	Alternative thoughts (%)	Emotion after (%)

Session 7 - Putting Thoughts and Ideas in Perspective (Phase 2)

Testing Alternatives (with behavioral tests)

Review client's condition and what has been learned (≈10 minutes)

- Follow-up on generalization attempts. Reinforce all efforts.
- If there was no attempt to generalize, take a few minutes to have the client examine an event that occurred between sessions and how the skill was relevant or applied.
- Follow-up on *Testing Alternatives* game. Do not pay too much attention to the labeling of emotions. Make the exercise a game of finding culturally relevant alternative interpretations. If the clients are having difficulties believing in the alternative thoughts, reassure that this it completely normal. It takes time to build self-confidence. Help them find facts that confirm their alternative interpretations and create behavioral tests where they can develop confidence in the new interpretations.
- For the behavioral tests, have the clients state their confidence in the belief to be tested and in the distress they expect to experience. Do it before and after the test to make it a relevant learning experience.
- Be mindful that clients may have experience significant adversity. It can be systemic or generational, and often from people saying they wanted to help. Do not question these issues. Leave space to express anger and grief and then focus on "here and now" situations that are amenable to cognitive restructuring.

Practicing Cognitive Strategies (Phase 2) in a Challenging VR context (≈40 min.)

- Use the *Practice Place* (with biofeedback) and the *Testing Alternatives* game sheet.
- Before starting the software, ask the client to identify from the menu which stressor to add to the virtual environment. Agree on a progressive sequence that will maximize emotional safety and the development of resilience skills.
- Guide clients in finding alternative thoughts on their own and check if these alternative views decrease the negative emotions.
- Complete the *Testing Alternatives* sheet for the client while they are immersed in VR and discuss it during or after the immersion.
- Accompany the clients in their quest for testing alternative thoughts. The idea is to look for alternative realistic thought, and develop the habit of testing these alternatives.
- Do not aim for a profound introspection and shared emotional experiences. Aim for a collaborative process where people learn by experience and reinforced successes.

Generalization and Closure of Session 7 (≈10 minutes)

- Show how you used the *Testing Alternatives* game during the immersion and invite the client to play the game to strengthen generalization. Do not make it a formal homework.
- For Session 7, the goal is to be able to restructure and test thoughts associated with perceived threat. Include behavioral tests to develop confidence in the alternatives.

Healing My Emotions Game 2: Testing Alternatives (in VR)

Situation	Thoughts (%)	Emotions (%)	Alternative thoughts (%)	Emotion after (%)

Session 8 – Inner-Dialogue Training

Review client's condition and what has been learned (≈10 minutes)

- Was the *Alternative Testing* game easy last week (because they already have good skills) or difficult (because they forgot, lacked the time, it wasn't clear, they could not problems to solve), and why?
- Follow-up on other attempts to generalize that have been set in past weeks and what you were supposed to discuss, if any. If more work is needed to feel a sense of mastery using a skill, guide the client on continued practice. Be mindful not to add too many skills to generalize to the client's personal schedule. Trying to learn too many strategies at the same time is more difficult than being able to use one strategy well.
- Decide if it is necessary to allocate today's session to cognitive restructuring instead of *Inner-Dialogue Training* (if you are not already behind in the program). *Session 9* is available as a buffer session, however all strategies of the program must be addressed.

Introduction to Inner-Dialogue Training (≈15 minutes)

- The value of *Inner-Dialogue Training* is that under some circumstances, *the best action we can do is talk to ourselves and go walk on the land*. Some people do indeed appreciate using grounding self-statements (e.g., in the case of flashbacks) and others prefer using motivational self-speech. However, to work at it's potential, the inner dialogue should be prepared in advance, <u>focus on resilience</u> (instead of anger or depression), and be <u>based on the individual's personal history and beliefs</u>.
- The idea is to tell oneself pre-learned personalized sentences (*Coping Self-Statements*) that have been found to be efficient in the past to build our own resilience.
- It is important to stay away from self-statements that are *only positive thoughts*. Life is not always positive and things don't always go well. Focus rather on personalized EMPOWERING thoughts (e.g., I know I can get through this. I can survive this. I am brave. I faced a bear last year, so I can certainly deal with this).
- Built with clients a <u>personalized</u> list of *Coping Self-Statements* they really believe in.
- These prepared self-statements can be repeated when resilience is needed.
- Coping Self-Statements can be used in 4 different contexts:
  1) Preparing to deal with a stressor
  2) Confronting and handling a current stressor
  3) Coping with the feelings of being overwhelmed
  4) Assessing efforts to manage emotions, and self-rewards
- Help the client find one or more *Coping Self-Statement* for each one of the four contexts above, and based on what they already tell themselves to cope.
- The lists on the following pages are examples to help psychotherapists. The individualized examples can be found while talking with the client before or during the immersion in VR.

Context 1: Preparing for stressor

Purpose:

- Focus on specific preparation for task
- Combat negative thinking
- Emphasize planning and preparation

Examples:

- “Here is a list of what I have to do…”
- “I can develop a plan to deal with it”
- “I will only think of what I can do about it”
- “This could be a rough situation, but I can face it”
- “I can work out a plan to handle this”
- “Remember, you stick to the issues and you don’t take it personally!”
- “Stop worrying! Worrying won’t help anything”
- “Here is a list of helpful things I can do instead of worrying…”
- “I am feeling tense – that’s normal and it is OK”
- “Maybe I am just eager to confront the situation!”

Context 2: Confronting and handling stressor
Purpose:

- Control stress reaction.
- Reassure that one can handle the situation.
- Reinterpret stress as something that can be used constructively.
- Reminder to use coping responses such as relaxation.
- Remain focused on the task or situation.

Examples:

- "I will breathe and stay focus – I can face this challenge!"
- "Let's go and stay focused".
- One step at a time.
- Just chunk the stress into manageable units.
- Don't think about my stress, just about what I have to do.
- This stress is what the trainer said I might feel.
- It is a reminder to use my coping exercises.
- This tenseness can be an ally, a cue to cope.
- Relax, I'm in control. Take a slow deep breath. Ah, good.
- As long as I keep my cool, I'm in control of the situation.
- Don't make more out of this than I have to.
- Look for positives, don't jump to conclusions.
- I have a lot of different coping techniques I can call upon.
- Things are not as serious as I make them out to be.
- I can just sit back and take it easy.

Context 3: Coping with Feelings of Being Overwhelmed

Purpose:

- Remind that this feeling does *not* always occur
- Set up contingency plans and get prepared for high levels of stress
- Prepare to deal with a worsening situation when feeling overwhelmed
- Encourage to remain in the situation until it resolves, if this outcome is likely
- Notice that it may be time to leave a situation that is likely to degenerate
- Stay focused on present instead of the future
- Accept feelings and wait for them to decrease
- Learn to notice elements of control when the situation worsens

Examples:

- Walking on the land is relaxing for me.
- When stress comes, just pause and practice relaxation
- "Keep my focus on the present and the task at hand"
- "Label my stress on a 0 to 10 scale and watch it change over time"
- "I should expect my stress to rise sometimes, but it will come down eventually."
- "I don't have to eliminate stress totally, only to keep it manageable"
- "My muscles are getting tight, so it is time to exhale and relax"
- "Let's slow down, I am reacting too fast and it is not helping"
- "Let's define the problem I am facing now"
- "Let's take the problem step by step and one at a time.
- "My stress is a signal that I need to act, not a problem"
- "It's time for problem solving"
- "Let's take a moment to ground myself and feel the land under my feet"
- "I can laugh at this situation, this usually works for me"
- "Let's take a moment to smell the air… and then stay focused"

Context 4: Assessing Efforts to Manage Emotions, and Self-Rewards Purpose:

- Coping self-statements can also be used after a stressor!
- It is a good idea to assess my attempt to be resilient
- It is important to learn lessons from what did not work
- Look back over past experiences to see what has been learned
- Recognize small steps, don't belittle gradual progress
- Praise oneself for trying!
- Remember the importance of keep trying and not expecting perfection

Examples:

- "It wasn't as bad as I expected".
- "It's getting better each time I use this procedure (maybe "method").
- "It didn't work, but that's OK".
- "Practice is everything…".
- "I can be happy with the progress I'm making".
- "I can't wait until I tell the others how it went".
- "I handled it pretty well".
- "Good, I did it. Next time I'll do even better".

Practicing Inner-Dialogue Training in Challenging VR context (≈25 minutes)

- *Coping Self-Statements* are personalized phrases that are meaningful and they will differ with each person. The statements in the boxes above are only examples! Find ones that are relevant and deeply believed by the client.
- Use the *Practice Place* (with biofeedback) to help clients to:
  - Increase their awareness that they can use coping self-statements when facing stressors.
  - Practice and remember their self-statements.
- Before starting the software, ask the client to identify from the menu which stressor to add to the virtual environment. Agree on a progressive sequence that will maximize emotional safety and the development of resilience skills.
- Look at the list of stressors from the menu.
- Accompany the client in the VR experience and discuss openly when and how to use their coping self-statements. They could say it out loud and in the language they use when they are talking to themselves (Inuktitut perhaps).
- Increase the stressors when necessary, as previously agreed with the client.
- Don't forget to guide the client using the coping statements in each of the four contexts, if possible.
- Coping self-statements must not be used to replace behaviorally testing hypotheses. They are to be used in situation where the only alternative is tolerate the situation until it is over.

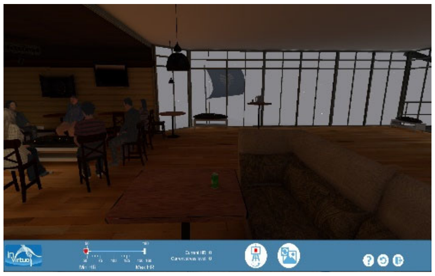
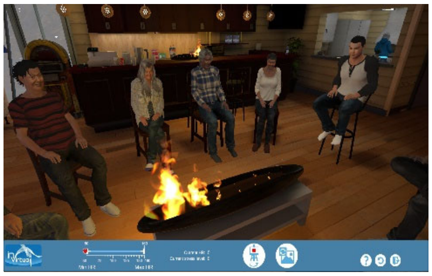
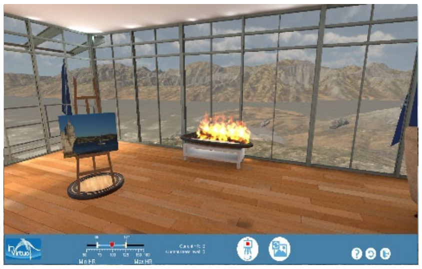

Generalization and Closure of Session 8 (≈10 minutes)

- Remind the client of the importance of practice to master skills.
- Always make sure the client understands the point of generalizing between sessions the skills that were developed today.
- For Session 8, the goal is to be confident when talking to oneself.
- Clients can carry and use reminders of their coping self-statements. Some client will carry a carved bone, a piece of paper or an object that is meaningful to them. It can also be a spiritual object.

Session 9: Buffer Session

Review client's condition and what has been learned (≈10 minutes)

- Follow-up on any previous attempt to generalize strategies. Assess their usefulness and sense of mastery from the client.
- Make sure the notion of testing beliefs, or behavioral experiments, is mastered. Cognitive techniques are used in this program to pinpoint situations that are perceived as threatening and engage in behaviors that will lead to the development of more functional alternative beliefs. Actions, or behavioral experiments, are the key to cognitive changes!

Your Option A (≈40 minutes): Completing the work already initiated

- This session is used as a buffer if more time was previously needed during the program to master some skills. For example, if four sessions were devoted to relaxation instead of three, it is now time to address material scheduled for *Session 8*. There is no other option for you than to proceed with covering all the content of the program.

Your Option B (≈40 minutes): Generalise the skills to other situations

- If there were no delays during the program, use the session to ask the client if there are other stressors that were not addressed in the program and to which he or she can generalize his or her skills. Take a moment to explore how to apply the EM skills practiced in this program to these newly identified stressors. Do not introduce new skills or therapeutic strategies.
- Then let the client decide if you are to either:
    - Apply the EM program to other stressors that have not been addressed before. This implies working in increasing flexibility in the use of EM and resilience skills to generalize to other personal life problems. The focus usually becomes less on anxiety and perceived threat, and more on other perceptions and emotions. But do not fully target problems that will require significantly more sessions to be improved (e.g., addiction, financial issues).
    - Stay with the initial stressors and repeat the content of a previous session to increase confidence in EM skills. The focus will not be on actually working on the newly identified stressors, if any. The decision implies that clients can generalize the program on their own with minimal guidance and time will be allocated to try to become better than the therapist on some EM skill.

Generalization and Closure of Session 9 (≈10 minutes)

- Set-up occasions to generalize as you see fit.

Session 10 – Putting all in practice for the long term

Review client's condition and what has been learned (≈10 minutes)

- Follow-up on any previous attempt to generalize strategies. Assess their usefulness and sense of mastery from the client. Highlight how behaviors can change beliefs and emotions.
- Introduce the concepts of therapy closure. The idea is to bring closure to what you experienced together during this program. The first step will be to discuss what they have learned (or already knew). It is important to focus on the notion that every improvement, big or small, is to be attributed to the client and not to you. One of the guiding principles of this program is that people <u>improved their resilience by their actions, and not by listening to a professional</u>.

Explore client's perception of intervention (≈10 minutes)

- Reinforce client's engagement in the program, their efforts and progresses.
- Empower clients with the new skills they've learned and make sure they had a positive experience in therapy.
- This portion of the program is likely to be based on a discussion between the two of you. If previous experience with this client revealed that they are more talkative when immersed in VR, than you can use an immersion in VR to facilitate discussion (e.g., in a location they liked in the VR environments).
- Sharing their wisdom with you, or with other people after treatment, is important.

Concepts of set-back versus recurrence: (≈10 minutes)

- Explain that discussing about set-backs does not mean you expect problems to recur and the program to fail in the long term. Discussing that it may be difficult to manage emotions in the future is not a fatality. Just like bringing out snowshoes does not mean it will snow; you are happy that you brought them if it does.
- The key to further improvements and maintaining gains includes three steps:
    - Finding high risks situations. By doing so, you would know to expect stress if they happen and to engage in using the skills you've learned to master.
    - The difference between a set-back in anxiety and the recurrence of an anxiety disorder. A set-back in anxiety is when you stumble, and the recurrence of an anxiety disorder is when the whole anxiety problem comes back again. Stumbling happens, but if we don't make a fuss about it and consider it as a source of information, it leads to better resilience. When we consider stumbling as a big catastrophe that takes power over us, it gets inflated and leads to discouragement and an actual recurrence of anxiety

disorders. To prevent this, it is important to soften the emotional significance of set-backs and consider them as sources of information.
    - Having a plan for what to do in case of set-backs in managing emotions.

Discuss concepts of EM in the long term while in a challenging context in VR: (≈15 minutes)

- Discuss with the client if you should use the *Snowy Place* or the *Practice Place* (without any additional stressors) to use as a safe place where the two of you worked together over previous challenges during the program. While the client walks in the environment, discuss more about long term efficacy based on what happened in VR in past sessions and how to appraise set-backs as normal fluctuation in emotions and stressors.

Elements of long term efficacy

- Identify internal and external risk factors and high-risk situations
- Consider a set-back as an opportunity for learning what went wrong
- Create a dynamic prevention plan to keep using what was useful in therapy

Generalization and Closure of Session 10: The Treasure of What Was Learned (≈15 minutes)

- The last therapy session is an occasion for the client to prepare a treasure to keep for themselves. This treasure can be a letter, a drawing or a handcraft. Audio recording are also acceptable, if they can remain confidential.
- Ask the client to create a "take home object" (bracelet, visual representation, etc.) that summarizes the three elements mentioned in the box above and discussed during the session. As a client, the idea is to prepare today something to refer to in case of future needs. If needed in the future, the client can go back to remind the key points learned. This personal treasure is to be kept personal and used if necessary.
- The formats proposed above are only suggestions and must be adapted to each client. Do not use words related to relapse prevention (lapse/relapse).
- Plan ahead with the client how and when the treasure will be done! You have 15 minutes to work on this occasion to generalise the intervention over time. Most of the time should be devoted to plan the preparation of the treasure and anticipate hurdles. The treasure will be useful only if it is done.

Delete from the computer all personal images from this participant. And empty the recycle bin of the computer.

Appendix 1 – Operating Protocol for Setting the Biofeedback Session

Materials

- Computer (1)
- TPS sensor (1)
- Oculus VR headset (1)

Software

- TPS.exe
- Oculus Application
- Virtual environments The *Snowy Place* and *The Practice Place*

Steps to follow / Instructions

These instructions below describe the steps to follow to perform a biofeedback session with VR and real-time data visualization on the web portal. Every step is done on the same computer. On the web portal, make sure a participant profile is created and sessions are assigned to them, otherwise, see *More Information* section.

1. Set up the VR headset (Head Mounted Display – HMD)
    1. Open Oculus application
    2. The VR headset must be connected to the computer and the inside light green
    3. The participant can now wear the HMD
2. Set up the web portal
    1. Execute the web portal
    2. Select the desired session
    3. On the session's page, select **Start Session**
    4. Confirm the session details by clicking on **Next Step**
    5. Follow the steps on the Start Session page
3. Set up the TPS Application
    1. Execute the TPS Application
    2. On the TPS Application form, copy & paste the session ID, enter the server address (localhost:8000) and the TPS serial number (ex. TP001234)
4. Set up the VR environment
    1. Execute the desired virtual environment (*Snowy Place* or *Practice Place*)
    2. On the menu, enter the username, the password and the session ID.
5. Execute
    1. On the web portal, select **Start Session**
    2. On the VR scene, select **Start**
    3. On the TPS Application, select **Connect**
6. Alternate the view (**Hit Alt Tab**) to see the Portal or the virtual environment.

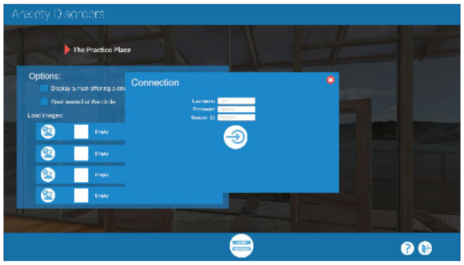

More Information

Create a Participant's Profile

1. Go to Patients or Admin page.
2. Select **+ Create User**
3. Filed the participant details *

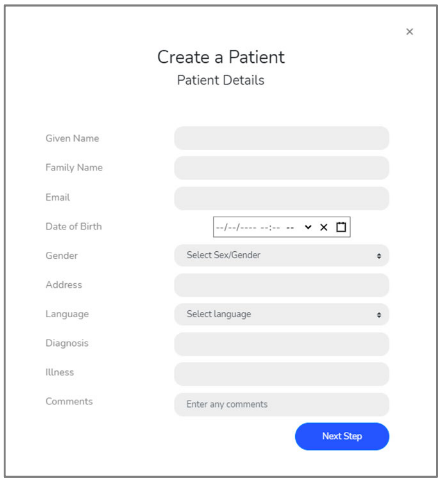

4. Select **Next Step**
5. Confirm the details by clicking on **Confirm**
6. Participant profile is now created

** The only fields required are Given Name, Family Name and Email. Email must be unique.*

Assign a Session Plan to a Participant

1. Go to Session Plans page
2. Select the desired session plan
3. Click on **+ Assign a Patient**

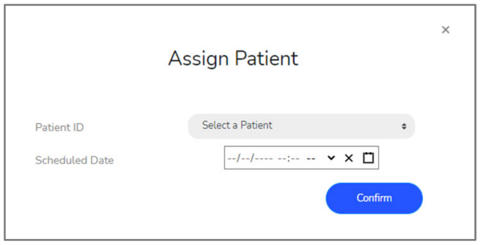

4. Select the participant
5. Select the start date for the first session
6. Click on **Confirm**
7. The sessions are now created for the participant

Appendix 2 – Operating Protocol for the Virtual Environments

To launch the virtual environments:

1. Click on the folder named Virtual Environments on the desktop.
2. Click on the shortcut file corresponding to the desired virtual environment (blue icon).

***The Snowy Place* :**

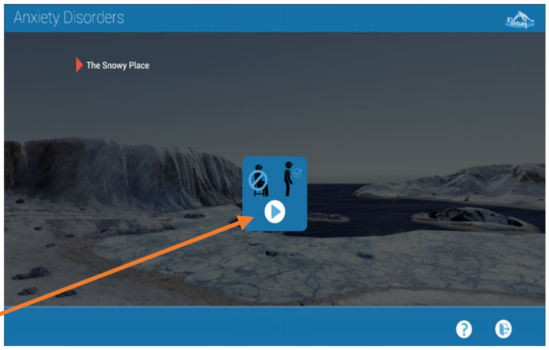

1. The menu on the right will be displayed to confirm this is the desired virtual environment and the **Launch** button will remind you the user must be standing during the immersion.
2. You will see at the bottom of your interface a button to **Quit** the immersion, and a **Help** button to access the treatment manual (see image at the bottom of the page). The same icons will be visible during the immersion. This information is not visible to the user during the immersion.
3. Click on the **Launch** button to start the immersion.
4. The user will be on the top of a hill. The user must use the small joystick button on the Oculus Touch to move forward and backward. To turn, the user must look in the desired direction and move forward.
5. It is possible to teleport the user by clicking on the icons representing three locations to save the effort of walking to these locations: the start-up point, near the caribous and at the eastern peninsula.

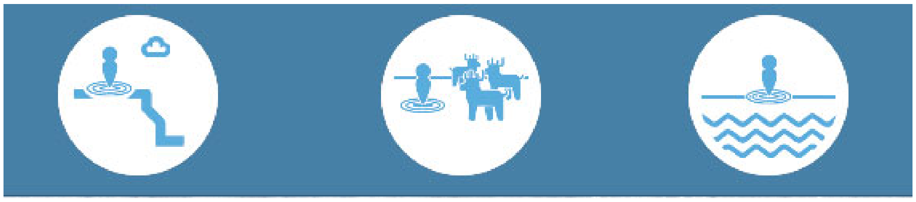

Illustration of the Help menu displayed on the screen

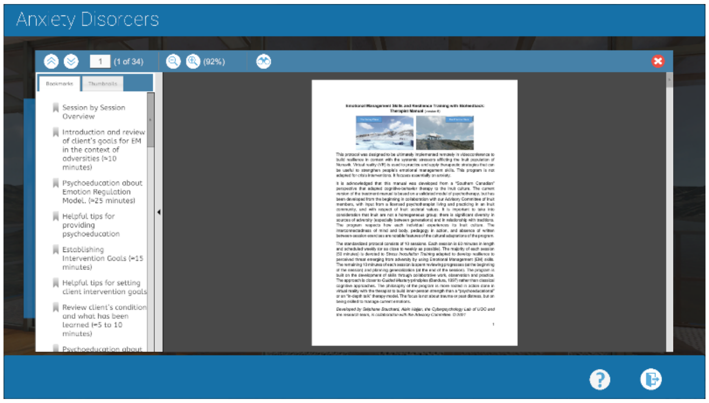

The Practice Place :

1. The menu on the right will be displayed to confirm this is the desired virtual environment and the **Launch** button will remind you whether the user must be standing or seated during the immersion.

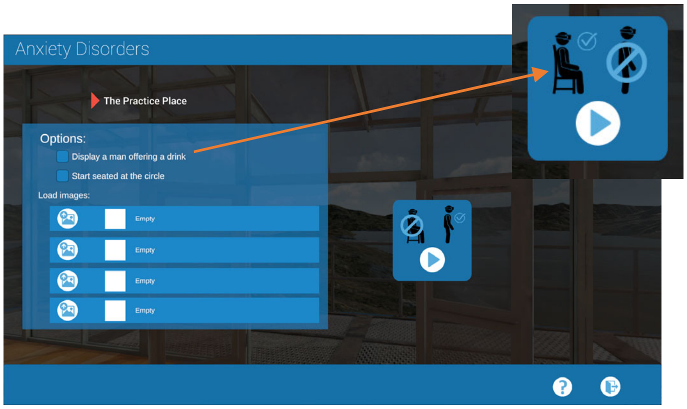

2. You will see at the bottom of your interface a button to **Quit** the immersion, and a **Help** button to access the treatment manual. The same icons will be visible during the immersion. This information is not visible to the user during the immersion.
3. You can select a few options:
   - A man standing up, looking at the user, and offering a drink from a cup can either be present or not in the virtual environment. The liquid in the cup is not visible and the cup could represent water, coffee, alcohol, etc.

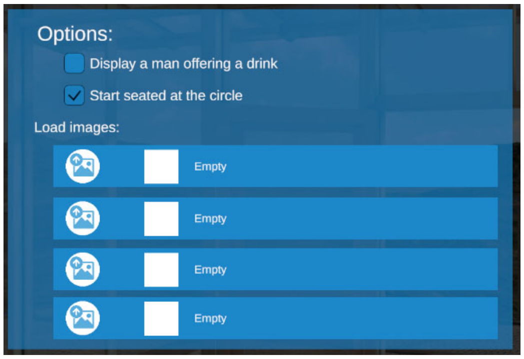

   - The user can be seated at the Talk Circle, or not. When the option Start seated is selected, the immersion will start with the user seated on a virtual chair and it is impossible to move away from the chair. In this case, the user must also by physically seated in your office. If the option is not selected, the chair will be left empty, the user will by physically standing in your office and able to move freely in the virtual environment. The empty chair may represent a chair left by someone missing from the group. The user can also walk to the chair and occupy this space to talk in the Talk Circle.
3. You can load up to four images that will be displayed on the easel in the virtual environment.

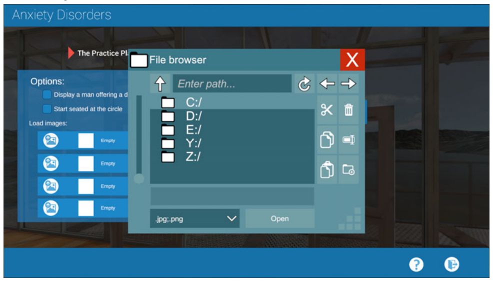

   a. The image files **must already be transferred** on the computer.
   b. You can select generic images (see Appendix 3) if the client did not bring personalized images.
   c. You can use personalized images either brought by the client, taken from your smartphone camera, or that you found on the web following the client's guidance. Personalized images

must be named according to the following convention: **ID number of the participant_a word describing the image** (e.g., 23_dogs.JPEG).

4. Click on the **Launch** button to start the immersion. The user can walk freely everywhere in the virtual environment.
5. The user must use the small joystick button on the Oculus Touch to move forward and backward. To turn, the user must look in the desired direction and move forward.
6. In the large space, the user can walk close or around an easel that will display up to five images. The easel will turn on itself and always face the user. To stop or restart the easel from rotating, click on the corresponding image.

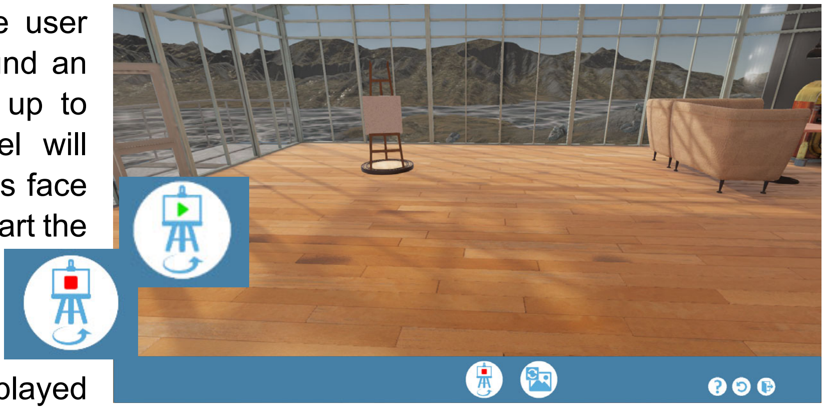

The default image displayed on the easel is a blank image. You can select to display any of the four images you could have loaded on Step 3 by **clicking on the other icon** in the bottom center: 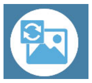 You will see the name and a snapshot of the file you selected. Just **click on the relevant image** to have it displayed on the easel.

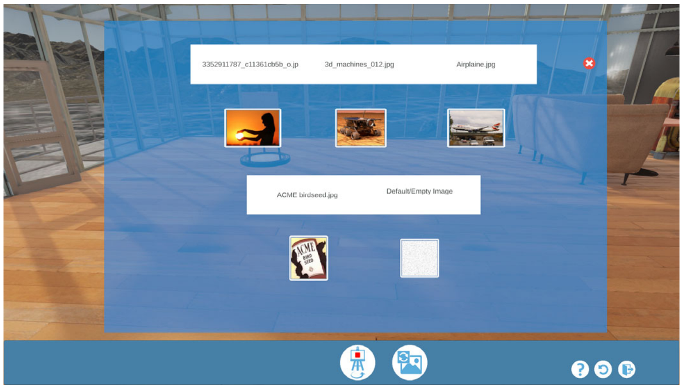

7. When you hit **Quit** the virtual environment, you will get a message stating that images will be cleared from the menu (but will remain on the hard drive). If you only want to restart the immersion without having to perform Step 3 again, click on No (the selected images will then remain available in the above menu). It is recommended to **always click Yes**, <u>unless</u> you only want to restart the immersion <u>with the same participant</u>. Clicking Yes will prevent occasions where participants could be exposed to personalized images from previous immersions with other participants.

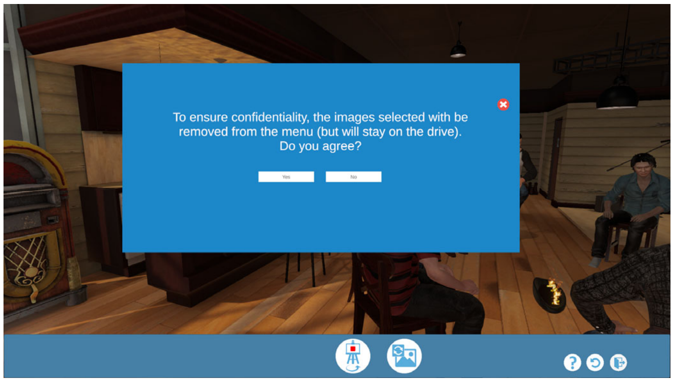

Images illustrating the biofeedback display

In *The Snowy Place*, the biofeedback is expressed by the intensity of the blizzard (clouds, snow, wind and reduced visibility).

Stress = 1 / 10 Stress = 2 / 10 Stress = 3 / 10 Stress = 4 / 10 Stress = 5 / 10

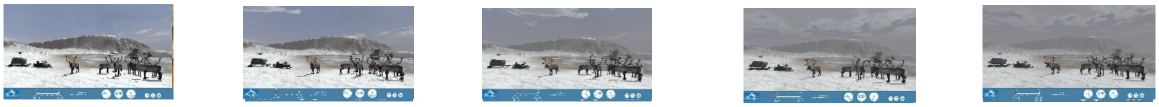

Stress = 6 / 10 Stress = 7 / 10 Stress = 8 / 10 Stress = 9 / 10 Stress = 10 / 10

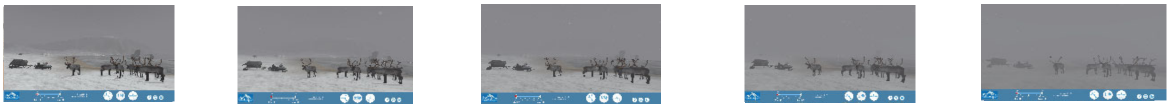

In *The Practice Place*, the biofeedback is expressed by the warmth / intensity of the many Qulliq and the intensity of the blizzard visible by the windows, as illustrated below.

Stress = 1 / 10 Stress = 2 / 10 Stress = 3 / 10 Stress = 4 / 10 Stress = 5 / 10

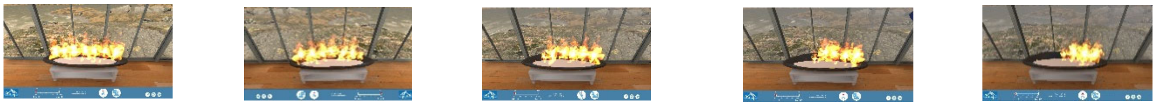

Stress = 6 / 10 Stress = 7 / 10 Stress = 8 / 10 Stress = 9 / 10 Stress = 10 / 10

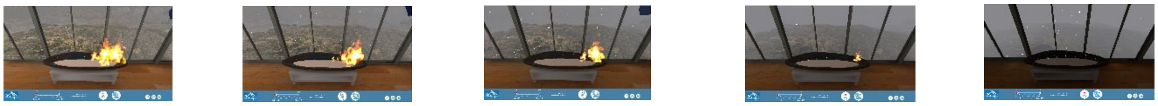

Stress = 1 / 10 Stress = 2 / 10 Stress = 3 / 10 Stress = 4 / 10 Stress = 5 / 10

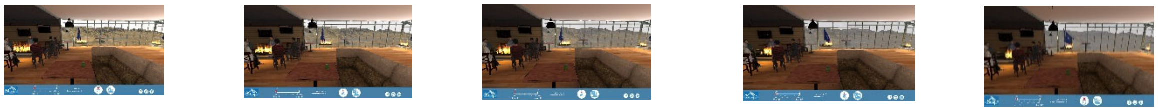

Stress = 6 / 10 Stress = 7 / 10 Stress = 8 / 10 Stress = 9 / 10 Stress = 10 / 10

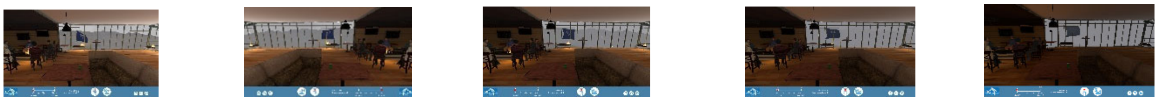

To adjust the sensitivity of the biofeedback, move the minimum and maximum values of the slider with the computer mouse. Moving only one end can guide the user to improve in the other direction (e.g., in the lower right image below, a HR of 100 would represent a lot of stress and a decreased in HR would be very noticeable).

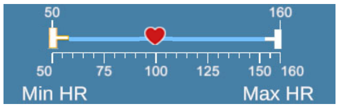

<- Less sensitive to variations in hear rate. HR has more room to change from Min (0%) to Max (100%).

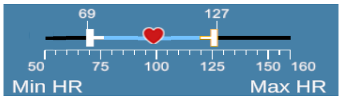

<- More sensitive to variations in heart rate. Small changes in HR represent larger change from Min (0%) to Max (100%).

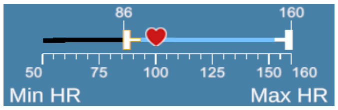

You can also make it more sensitive by moving only the Min or Max values

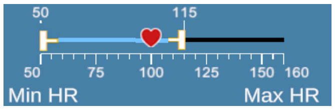

Appendix 3 – List of Generic Images

Images have been selected from Internet before starting the trial and may be used if they are considered useful by some client. Some of these stimuli were mentioned by the Advisory Committee as potentially interesting to include in the virtual environment. The suggestions were proposed when considering the possibility to create digital versions of these stressors and include them in the *Practice Place*. These specific images have not been approved by any instance. Other images have been added since then.

All Images were publicly available from the Internet but are most likely protected by copyrights. They cannot be distributed outside the context of this clinical trial.

Some images may be traumatizing for some people. You must first discuss with the client which kind of image would be suitable. You can then show them the image on the computer screen before selecting it for the treatment.

Images are organized in the following folders:

- Accident
- COVID 19
- Dog sled
- Montreal
- Permafrost and houses
- Polar bear
- Police
- Press event

The following pages cannot be distributed or shared to protect copyrights.

Some of the following images may trigger emotional reactions in some people.
